# Biliary atresia and cholestasis plasma non-targeted metabolomics unravels perturbed metabolic pathways and unveils a diagnostic model for biliary atresia

**DOI:** 10.1038/s41598-024-66893-2

**Published:** 2024-07-09

**Authors:** Bang Du, Kai Mu, Meng Sun, Zhidan Yu, Lifeng Li, Ligong Hou, Qionglin Wang, Jushan Sun, Jinhua Chen, Xianwei Zhang, Wancun Zhang

**Affiliations:** 1 Health Commission of Henan Province Key Laboratory for Precision Diagnosis and Treatment of Pediatric Tumor, Children’s Hospital Affiliated to Zhengzhou University, Zhengzhou, 450018 China; 2 Henan Key Laboratory of Rare Diseases, Endocrinology and Metabolism Center, The First Affiliated Hospital, and College of Clinical Medicine of Henan University of Science and Technology, Luoyang, 471003 China; 3 Henan Key Laboratory of Children’s Genetics and Metabolic Diseases, Children’s Hospital Affiliated to Zhengzhou University, Zhengzhou, 450018 China; 4 Henan International Joint Laboratory for Prevention and Treatment of Pediatric Disease, Children’s Hospital Affiliated to Zhengzhou University, Zhengzhou, 450018 China; 5 Department of Pharmacy, Affiliated Cancer Hospital of Zhengzhou University, Henan Cancer Hospital, Zhengzhou, 450008 China

**Keywords:** Metabolomics, Biliary atresia, Cholestasis, Blood plasma, Diagnostic model, Gastrointestinal diseases, Diagnostic markers

## Abstract

The clinical diagnosis of biliary atresia (BA) poses challenges, particularly in distinguishing it from cholestasis (CS). Moreover, the prognosis for BA is unfavorable and there is a dearth of effective non-invasive diagnostic models for detection. Therefore, the aim of this study is to elucidate the metabolic disparities among children with BA, CS, and normal controls (NC) without any hepatic abnormalities through comprehensive metabolomics analysis. Additionally, our objective is to develop an advanced diagnostic model that enables identification of BA. The plasma samples from 90 children with BA, 48 children with CS, and 47 NC without any liver abnormalities children were subjected to metabolomics analysis, revealing significant differences in metabolite profiles among the 3 groups, particularly between BA and CS. A total of 238 differential metabolites were identified in the positive mode, while 89 differential metabolites were detected in the negative mode. Enrichment analysis revealed 10 distinct metabolic pathways that differed, such as lysine degradation, bile acid biosynthesis. A total of 18 biomarkers were identified through biomarker analysis, and in combination with the exploration of 3 additional biomarkers (LysoPC(18:2(9Z,12Z)), PC (22:5(7Z,10Z,13Z,16Z,19Z)/14:0), and Biliverdin-IX-α), a diagnostic model for BA was constructed using logistic regression analysis. The resulting ROC area under the curve was determined to be 0.968. This study presents an innovative and pioneering approach that utilizes metabolomics analysis to develop a diagnostic model for BA, thereby reducing the need for unnecessary invasive examinations and contributing to advancements in diagnosis and prognosis for patients with BA.

## Introduction

Biliary atresia (BA) is a cholestatic disorder characterized by the obstruction of both intrahepatic and extrahepatic bile ducts, primarily affecting infants aged below 3 months. The disease is characterized by progressive inflammation and fibrosis of the biliary system, which can lead to significant mortality in affected children before the age of 2 if surgical intervention is not pursued. This poses a formidable threat to their survival^1–3^. The incidence of BA is approximately 1 in 10,000 cases worldwide, accounting for 30% of all infants presenting with cholestasis (CS). Moreover, BA represents the most prevalent etiology leading to liver transplantation in children diagnosed with end-stage liver disease in developed countries. Due to the overlapping clinical manifestations between BA and CS, routine examinations encounter challenges in detection, with less than 2% of BA patients exhibiting abnormalities through auxiliary tests; thus, clinical differential diagnosis remains a formidable task^4–8^. However, children diagnosed with BA often face an unfavorable prognosis and compromised quality of life, necessitating prompt surgical intervention. The diagnostic challenges associated with this condition frequently lead to delayed identification and missed opportunities for optimal surgical timing, ultimately resulting in diminished survival rates^9^. Therefore, diagnosis of BA is imperative to optimize surgical outcomes and long-term survival rates in pediatric patients with BA. Currently, clinical diagnostic modalities for BA encompass ultrasound examination, duodenal fluid analysis, and radionuclide hepatobiliary imaging^10,11^. Among these methods, laparoscopic or laparotomy cholangiography is considered the gold standard for diagnosing BA^12^, but all are invasive procedures. However, during the early stages of the disease, parental acceptance tends to be suboptimal, resulting in delays in both disease diagnosis and treatment. In conclusion, the development of a precise and non-invasive diagnostic model for BA holds significant value in terms of facilitating detection and improving prognostic outcomes for patients with BA.

The emergence of metabolomics has brought about novel perspectives for disease diagnosis, which is noteworthy. Metabolomics aims to comprehensively characterize small molecules within a sample, enabling accurate identification and quantification of in vivo metabolites with molecular weights less than 1.5 KDa^13^. This precise reflection of the biological metabolic characteristics associated with disease states facilitates an enhanced comprehension of pathophysiological processes during disease progression, thereby enabling the development of biomarker profiles for specific diseases or conditions and the discovery of novel biomarkers for disease diagnosis^14,15^. Jiali Qin et al.^16^ conducted a metabolomics analysis to compare the metabolic differences between follicular thyroid carcinoma and follicular thyroid nodules, revealing distinct metabolite profiles in these 2 groups. Notably, 6 lipid compounds, including lysophosphatidic acid, were identified as potential biomarkers. Additionally, Yuan Tian et al.^17^ conducted a metabolomics analysis of colorectal cancer tissues and their adjacent non-involved tissues, revealing distinct metabolic phenotypes associated with colorectal cancer. These tissue-specific metabolic profiles not only differentiate colorectal cancer tissues from their adjacent non-involved tissues but also discriminate between low-grade and high-grade tumor tissues with high sensitivity and specificity, highlighting the significance of metabolomic phenotyping as a valuable diagnostic tool for tumors. Moreover, Filipe Tenorio Lira Neto et al. developed a metabolomics model that effectively discriminates between varicocele infertile men and varicocele fertile men, achieving accuracies of 94.64% and 100%, respectively^18^. Therefore, metabolomics analysis holds significant application value in the field of BA, enabling the identification of alterations in metabolic pathways and biomarkers associated with BA, thereby facilitating the establishment of a diagnostic model for BA.

In this study, we performed metabolomics analysis on clinical plasma samples obtained from 90 patients diagnosed with BA, 48 patients diagnosed with CS, and 47 healthy individuals serving as normal controls (NC). Our primary aim was to investigate distinct metabolites and aberrant pathways associated with BA, while also developing diagnostic models based on potential biomarkers. The main contributions of this study include: (1) a comprehensive evaluation of the metabolomic differences among BA, CS, and normal controls (NC) through systematic analysis; and (2) the development of a metabolomics-based diagnostic model for BA. The establishment of this diagnostic model holds significant promise in facilitating surgical intervention and accurate diagnosis in cases of BA.

## Materials and methods

### Moral approval

This study received approval from the Ethics Committee of Henan Children's Hospital (2022-H-K07), and informed consent was obtained from all patients for sample collection. All procedures were conducted in strict adherence to relevant guidelines and regulations.

### Sample collection

A total of 185 plasma samples (90 cases of BA, 48 cases of CS, and 47 cases of NC) were collected and processed at Henan Children's Hospital between October 2018 and January 2022. Inclusion criteria: (1) Confirmed by surgical intervention or clinical diagnosis, meeting the diagnostic criteria of BA and CS; (2) Comprehensive clinical and laboratory data were available; (3) Informed consent was obtained; (4) Samples from BA and CS patients were taken from fasting plasma on the morning of the operation; (5) The NC plasma samples were taken from the fasting plasma of patients on the morning. Exclusion criteria: (1) BA or CS cannot be clearly diagnosed clinically (2) incomplete clinical data in children; (3) refusal of informed consent by guardians. Plasma samples were collected and were immediately frozen at − 80 °C for metabolomics analyses.

### Metabolomics analysis via high performance liquid chromatography-mass spectrometry (HPLC-MS)

The plasma samples were retrieved from storage at − 80 °C and promptly thawed in a refrigerator set at 4 °C. After vortexing for 10 s, 150 μL of plasma was transferred to a microcentrifuge tube with a capacity of 1.5 mL, followed by the addition of 450 μL acetonitrile maintained at 4 °C. Subsequently, the mixture was vigorously vortexed for 5 min at a speed of 3000 r/min and then subjected to centrifugation at 13,000 r/min for 15 min (at a temperature of 4 °C). Subsequently, a careful extraction process yielded approximately 300 μL of supernatant volume. To ensure the stability of the overall experimental results, quality control (QC) samples were prepared by combining equal amounts of supernatant from all samples. The samples were analyzed using a simple random sampling method after being randomly sorted, in order to mitigate potential systematic errors introduced by the sample processing sequence.

For the analysis of all extracts, an Agilent 6210 time-of-flight MS system equipped with an Agilent 1100 HPLC, a photodiode array detector, and a high-resolution-time-of-flight-MS with an electrospray ionization source was employed. The chromatographic separation was performed on an Agilent Poroshell 120 EC—C18 (2.7 μm, 3.0 × 100 mm) column. The metabolomics data were acquired under the following conditions: a mobile phase composed of A = 0.1% formic acid in water and B = 0.1% formic acid in acetonitrile, with elution conditions as follows: from 0 to 3 min, a gradient from 5 to 60% B; from 3 to 25 min, a gradient from 60 to 90% B; from 25 to30 min, a gradient from 90%to100%B; and finally, a constant flow of pure solvent B for the remaining time (30–40min). Experimental conditions included an injection volume of 10 μL, column temperature maintained at a constant value of 30 °C, and a flow rate set at a steady rate of 0.3 mL/min. Mass spectrometry analysis was conducted in both negative and positive ionization modes using nitrogen as the drying gas at approximately 325 °C with a flow rate set at 12 L/min and atomization pressure maintained at 35 psi. The capillary voltage was adjusted to 4,000 V for positive mode and 3,500 V for negative mode, while the fragmentation voltage was set to 215 V for positive mode and 175 V for negative mode with the separator voltage fixed at 60 V. The mass acquisition range encompassed all negative ions within the range of 0.05–1.5 KDa. The samples underwent HPLC–MS analysis to obtain the raw data files. Agilent Masshunter HPLC–MS software was employed for converting the original data files into a standardized format. XCMS software package, implemented on the R language platform, was utilized for retention time (RT) calibration, peak identification, noise filtration, and peak matching of the acquired mzData format files. Moreover, it facilitated the establishment of permissible deviations for both mass-to-charge ratio and RT (mass/charge ratio tolerance = 0.025 Da; RT tolerance = 0.5 min). Metabolites exhibiting a RT deviation of 0.5 min and a mass number deviation of 0.025 Da were considered as identical metabolites. Subsequently, a data matrix comprising mass/charge ratio, RT, peak area, and other pertinent information was obtained. Metabolite identification involved the utilization of both primary and MS techniques. Initially, the acquired primary MS information underwent targeted secondary MS analysis to obtain supplementary MS data that served as a reference for subsequent qualitative analysis. Furthermore, by leveraging the precise mass numbers of excimer ions such as [M^+^H]^+^ ions and high-resolution target MS/MS spectra in conjunction with observed fragmentation patterns across various metabolites, potential structures for differential metabolites were deduced through comprehensive analyses involving online databases (METLIN: http://metlin.scripps.edu/, HMDB: http://hmdb.ca/) as well as literature retrieval methods. Metabolomics analysis was conducted using MetaboAnalyst (https://www.metaboanalyst.ca/MetaboAnalyst/home.xhtml), which encompassed principal component analysis (PCA), partial least-squares discrimination analysis (PLS-DA), heatmap generation, volcano plot visualization, enrichment analysis, and identification of biomarkers. The PLS-DA integrates regression models and dimensionality reduction techniques to classify samples by establishing a linear relationship between the predictor and response variables. A heatmap is a graphical representation method for displaying a data matrix, where data values are depicted using a color gradient that reflects their magnitude. A volcano plot is commonly employed in differential experiments, particularly in metabonomics studies, to illustrate differential expression patterns. It utilizes fold change on the x-axis, statistical significance on the y-axis, and distinct colors to represent up- or down-regulation. Enrichment analysis is a statistical method utilized to ascertain whether a specific function or pathway exhibits an overrepresentation in a given list of metabolites.

### The construction of a diagnostic model for BA

We established the test set and validation set through random sampling, ensuring that the number of instances in our validation set was approximately half of the total to ensure representative characteristics. Based on our metabolomics data, we employed SPSS logistic regression analysis to construct the regression equation. A validation set comprising 42 BA cases and 24 CS cases was utilized for model validation, and subsequently, the prediction sensitivity and specificity of the model were calculated. Data processing was performed using IBM Corp.'s SPSS software version 22.0 (Armonk, New York), while Origin 2021 was employed for visualization.

### Ethics approval

This study was reviewed and approved by the committees of Henan Children's Hospital (2022-H-K07).

## Results

**Scheme 1:**
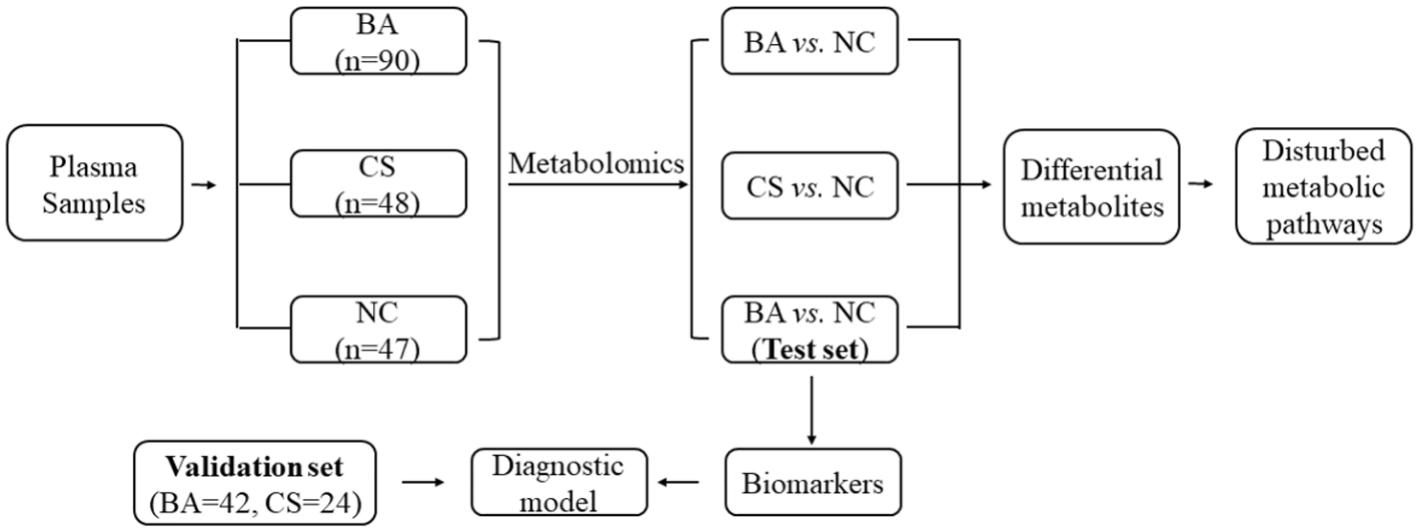
Outline of research method.

The general concept of this study is illustrated in Scheme 1. Metabolomics analysis was conducted on a total of 185 plasma samples, comprising 90 cases of BA, 48 cases of CS, and 47 cases of NC. Through the utilization of PCA, PLS-DA, heatmap visualization, volcano plot representation, biomarker analysis, enrichment analysis for data processing and interpretation, a diverse array of differential metabolic pathways associated with BA were identified. Ultimately, an innovative diagnostic model was established to facilitate detection and diagnosis of BA.

Initially, we conducted a retrospective analysis of patient clinical data and subsequently present the fundamental characteristics of the collected samples in Table S1. The results indicate that there were no statistically significant differences observed in the variables of age, male ratio, total bile acids (TBA), total bilirubin (TBIL), direct bilirubin (DBIL), alanine aminotransferase (ALT), and alanine aminotransferase (AST) between BA and CS samples. However, a significant difference was found in gamma glutamyl transferase (GGT) levels between the 2 groups.

### The Metabolomic differences among BA, CS, and NC

**Figure 1:**
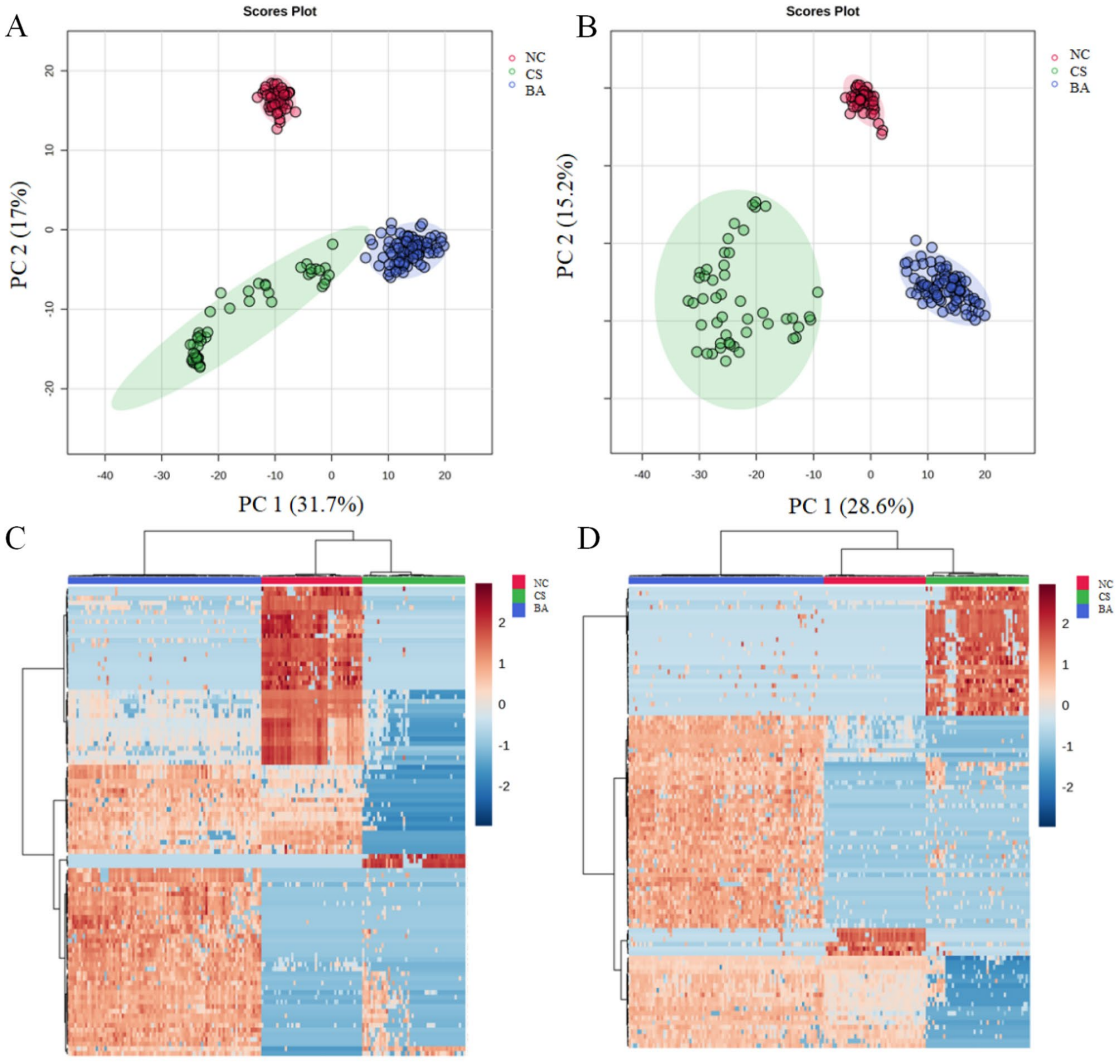
Plasma metabolomics of BA, CS and NC. Comparison of BA, CS and NC in PCA plot in positive mode (**A**) and in negative mode (**B**). The heatmap shows clear distinction of metabolites of BA, CS and NC patients in positive mode (**C**) and in negative mode (**D**).

The PCA plot shows the close clustering of QC samples under positive and negative modes, which verifies the robustness of our results (Fig. S1). The overall metabolite differences among the BA, CS, and NC groups were explored through plasma metabolomics analysis using untargeted metabolomics methods. PCA plots in both positive and negative modes (Fig. 1A and B) clearly demonstrated significant separation of the 3 groups under PCA analysis. Additionally, the heatmaps depicted metabolites with notable differences between the 3 groups in both positive and negative modes (Fig. 1C and D). Therefore, the metabolomics data revealed significant distinctions among the BA, CA and NC groups.

### Cross-comparison between BA, CS, and NC metabolomics groups

**Figure 2:**
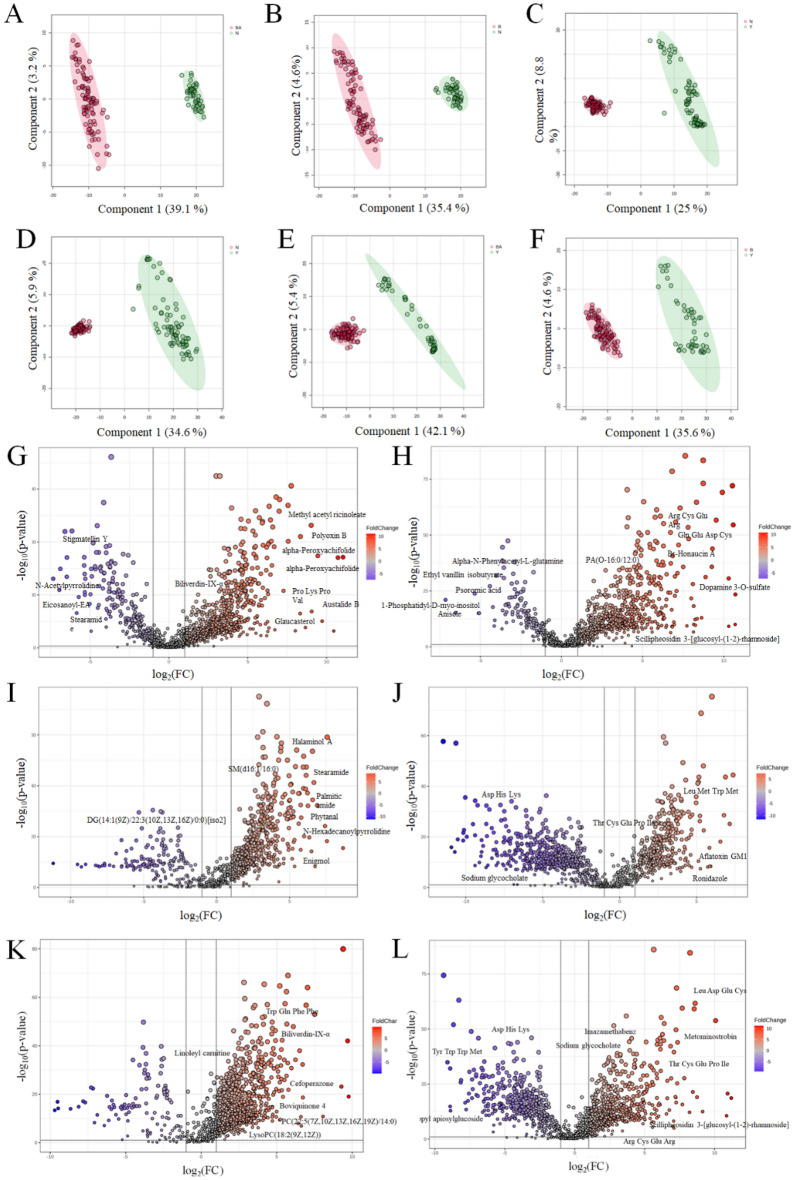
Metabolomics cross-group comparison of BA, CS and NC. The PLS-DA results for the comparison between BA and NC in positive (**A**) and negative modes (**B**), CS and NC in positive (**C**) and negative modes (**D**), BA and CS in positive (**E**) and negative modes (**F**). Volcano plot of metabolite of BA vs*.* NC in positive mode (**G**) and negative mode (**H**), CS vs*.* NC in positive mode (**I**) and negative mode (**J**), BA vs*.* CS in positive mode (**K**) and negative mode (**L**).

To gain a deeper understanding of the distinctions between BA, CA, and NC groups, cross-group comparisons were conducted using PLS-DA analysis to characterize the metabolic abnormalities between BA and NC, CS and NC, as well as BA and CS. Notably significant differences were observed in both positive and negative modes. Specifically, the cumulative R2 and Q2 values for BA *vs.* NC in the positive mode were 0.996 and 0.986 respectively (Fig. 2A, Fig. S2A). Similarly, in the negative mode, the cumulative R2 and Q2 values for BA *vs.* NC reached 0.999 and 0.989 (Fig. 2B, Fig. S2B). In terms of the comparison between CS and NC in the positive mode, a highly significant cumulative R2 value of 0.999, along with an excellent Q2 value of 0.991, was obtained (Fig. 2C, Fig. S2C). Similarly, in the negative mode analysis, CS vs*.* NC exhibited significantly high cumulative R2 (0.999) and Q2 (0.988) values (Fig. 2D, Fig. S2D). Furthermore, when comparing BA with CS in positive mode, we observed a remarkably high cumulative R2 value of 0.998 alongside an impressive Q2 value of 0.980 (Fig. 2E, Fig. S2E). Last but not least, the cumulative R2 and Q2 values for BA *vs.* NC in negative model are found to be exceptionally strong at 0.998 and 0.983 respectively (Fig. 2F, Fig. S2F). We subsequently conducted a comprehensive analysis of the differential metabolites using volcano plots. The volcano plots were utilized to depict the metabolites detected in both positive and negative modes for BA and NC (Fig. 2G and H), CS and NC (Fig. 2I and J), as well as BA and CS (Fig. 2K and L). Metabolites exhibiting a |Log_2_ fold change|> 1 with a significance level of *P* < 0.05 in the volcano plot were identified as differential metabolites. Therefore, our PLS-DA analysis revealed significant group differences and multiple differential metabolites were identified through volcano plots.

### Differential metabolites compared among the BA, CS, and NC groups

**Figure 3:**
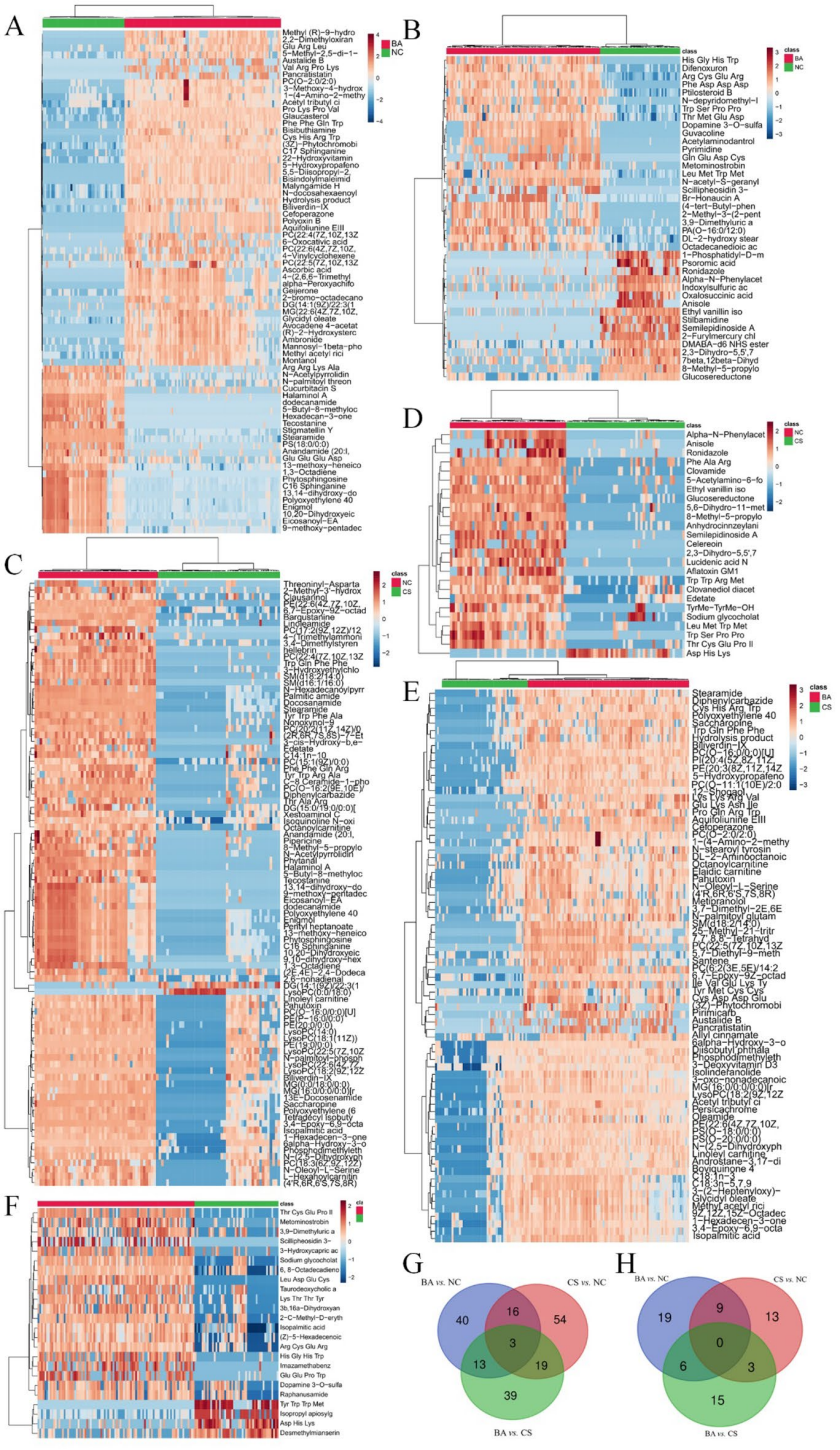
The altered metabolites in metabolomics. The heatmaps of metabolite of BA vs*.* NC in positive mode (**A**) and negative mode (**B**), CS *vs.* NC in positive mode (**C)** and negative mode (**D**), BA vs*.* CS in positive mode (**E**) and negative mode (**F**). The Venn plot in in positive mode (**G**) and negative mode (**H**).

In order to provide a more comprehensive depiction of the metabolic disparities between BA, CS and NC, we initially conducted a cluster analysis on plasma metabolites using compound correlation. The results were visually represented through a heatmap (Fig. S3), effectively highlighting the variations in metabolite expression across different groups. In addition, to further visualize the differential metabolites and demonstrate expression levels in each sample, a correlation-based heatmap was generated for the differential metabolites (Fig. 3). In the heatmap comparing BA and NC, we identified 72 differential metabolites in the positive mode, comprising 48 up-regulated metabolites and 24 down-regulated metabolites (Fig. 3A, Table S2). Additionally, we detected 40 differential metabolites in the negative mode, including 24 up-regulated metabolites and 16 down-regulated metabolites (Fig. 3B, Table S3). Similarly, in the volcano plot comparing CS and NC, we observed a total of 92 differential metabolites in the positive mode with predominantly up-regulation (91 up-regulated *vs.* 1 down-regulated) (Fig. 3C, Table S4). Furthermore, we found 25 differential metabolites in the positive mode for this comparison, consisting of 23 up-regulated metabolites and only 2 down-regulated ones (Fig. 3D, Table S5). Lastly, when examining BA *vs.* CS through a volcano plot approach, all of the identified 74 differential positive mode metabolites were found to be upregulated (Fig. 3E, Table S6) while among the negative mode differentials there were 20 upregulated compounds alongside 4 that showed decreased levels (Fig. 3F, Table S7). Furthermore, we employed a Venn diagram to investigate the shared differential compounds among the 3 groups. Remarkably, in the positive ion mode, 3 metabolites were consistently observed across all groups (Fig. 3G): Biliverdin-IX-α, Stearamide, and Polyoxyethylene 40 monostearate. Conversely, no common differential metabolites were identified in the negative mode (Fig. 3H). Therefore, the heatmaps revealed upregulation of metabolites such as LysoPC(18:2(9Z,12Z)), while metabolites including Asp His Lys exhibited downregulation. These findings suggest a significant correlation between these metabolites in BA and CS.

### Differential metabolic pathways compared among the BA, CS, and NC groups

**Figure 4:**
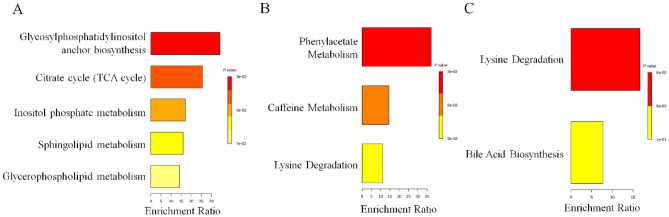
The altered pathways in metabolomics. The enrichment analysis of differential metabolism revealed distinct metabolic alterations among BA and NC (**A**), CS and NC (**B**), BA and CS (**C**).

The identification of abnormal metabolic pathways was facilitated by the discovery of abnormal metabolites, with separate enrichment analysis conducted among the groups. Enrichment analysis revealed the following results: In the BA vs*.* NC group, based on the 112 most significantly altered metabolites, our enrichment analysis demonstrated significant enrichment in glycosylphosphatidylinositol-anchor biosynthesis, citrate cycle (TCA cycle), inositol phosphate metabolism, sphingolipid metabolism and glycerophospholipid metabolism (Fig. 4A). In the CS vs*.* NC group, based on the 117 most significantly altered metabolites, we observed significant enrichment in phenylacetate metabolism, caffeine metabolism and lysine degradation (Fig. 4B). Moreover, in the BA vs. CS group, analysis of the 98 most significantly altered metabolites revealed a notable enrichment in lysine degradation and bile acid biosynthesis (Fig. 4C). The table presenting the metabolites corresponding to the enrichment pathways is displayed in Table S8. In summary, enrichment analysis revealed 10 metabolic pathways exhibiting significant alterations, thereby enhancing our understanding of aberrant pathways in BA and CS.

### Classification of BA and CS with selected candidate biomarkers

**Figure 5:**
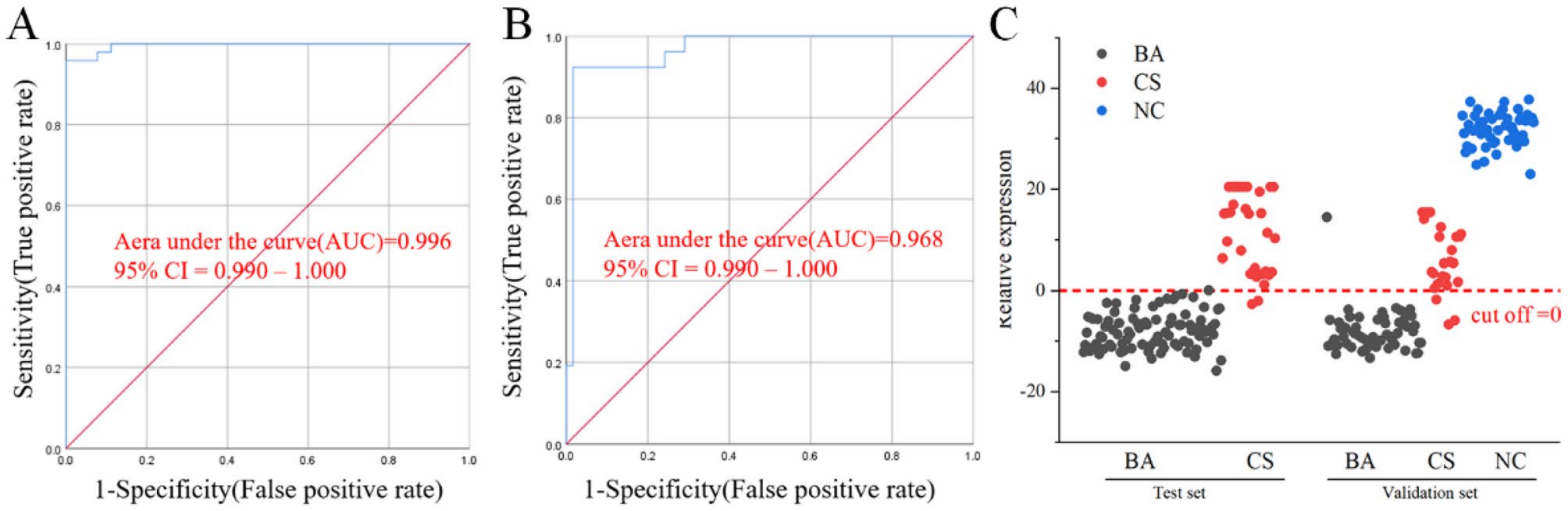
Establishment of BA diagnosis model. AUC value for prediction of BA based on 3 plasma differential metabolites (LysoPC(18:2(9Z,12Z)), PC(22:5(7Z,10Z,13Z,16Z,19Z)/14:0), and Biliverdin-IX-α) in test set (**A**) and validation set (**B**). The prediction accuracies in test set and validation set (**C**).

In order to gain a deeper understanding of the metabolic disparities between BA and CS, as well as to identify effective biomarkers that can serve as diagnostic tools for BA, our focus was directed towards conducting a comprehensive biomarker analysis comparing BA and CS samples. Through meticulous biomarker analysis, we successfully identified a total of 9 potential biomarkers in the positive mode (Fig. S4) and 9 potential biomarkers in the negative mode (Fig. S5), all exhibiting an area under the ROC curve greater than 0.8. The diagnostic model was constructed based on the potential biomarkers. However, our findings demonstrate that individual biomarkers exhibit limited detection performance, whereas a combination of these biomarkers yields superior detection performance. Therefore, based on our findings, we extensively reviewed relevant literature pertaining to BA and CS, and systematically explored various combinations. Ultimately, a diagnostic model was established by integrating LysoPC(18:2(9Z,12Z)), PC(22:5(7Z,10Z,13Z,16Z,19Z)/14:0), and Biliverdin-IX-α. Consequently, a diagnostic model was developed by integrating 3 biomarkers (LysoPC(18:2(9Z,12Z)), PC(22:5(7Z,10Z,13Z,16Z,19Z)/14:0), and Biliverdin-IX-α) using logistic regression analysis. The resulting regression equation is as follows: *Y* = 45.23 – 1.386*X*_1_ (LysoPC(18:2(9Z,12Z))) – 6.978 *X*_2_ (PC (22:5(7Z,10Z,13Z,16Z,19Z)/14:0)) – 1.614 *X*_3_ (Biliverdin-IX-α). The diagnostic model demonstrated a sensitivity of 95.8% and specificity of 98.9% in the test set. ROC analysis revealed an exceptional area under the curve (AUC) value of 0.996 (Fig. 5A), with a cutoff value set at 0 (Fig. 5C). In the validation set, the diagnostic model exhibited a sensitivity of 92.3%, specificity of 98.4%, and achieved an AUC value of 0.968 in ROC analysis (Fig. 5B), thus confirming its efficacy for predicting plasma risk classification in BA patients using LysoPC(18:2(9Z,12Z)), PC(22:5(7Z,10Z,13Z,16Z,19Z)/14:0), and Biliverdin-IX-α as viable biomarkers in combination. This approach holds promise for non-invasive detection of BA.

## Discussion

Portojejunostomy (Kasai procedure) currently represents the primary surgical approach for the management of BA. Increasing evidence suggests that performing the Kasai procedure at a younger age is associated with enhanced postoperative resolution of jaundice, improved native liver survival rate, and overall survival rate^19,20^. Consequently, timely detection and intervention are crucial in the diagnosis and treatment of BA. Although various diagnostic methods for BA exist, such as laparoscopy or cholangiography under open exploration, which is considered the gold standard^12^, all of these approaches are invasive procedures, and there is currently no non-invasive diagnostic technology available to effectively differentiate BA from CS caused by other etiologies. With the advancement of metabolomics research, serum metabolites have emerged as a novel diagnostic indicator for diseases. In this study, we conducted a comprehensive analysis of plasma metabolomic composition in patients with BA, aiming to differentiate BA from CS by identifying highly sensitive and specific metabolites using LC/MS technology. Subsequently, we identified multiple differential metabolites associated with BA and selected 3 potential biomarkers to construct a diagnostic model. Notably, in the validation set, our model achieved an impressive area under the receiver operating characteristic curve of 0.920.

As the liver plays a pivotal role in amino acid metabolism, it is imperative to investigate the metabolite profiles of BA and CS. Our liver function tests revealed no significant difference in serum ALT and AST elevation between BA and CS patients. However, there exists a distinct set of amino acid concentrations between these two groups. The heatmap analysis demonstrated that Trp Gln Phe Phe, Cys His Arg Trp, Tyr Met Cys Cys, Cys Asp Asp Glu, Lys Lys Arg Val, Ile Val Glu Lys Tyr, and Glu Lys Asn Ile expression were significantly increased in positive mode differential metabolites while Thr Cys Glu Pro Ile, Leu Asp Glu Cys, Arg Cys Glu Arg, Lys Thr Thr Tyr, Glu Glu Pro Trp and His Gly His Trp expression were significantly increased in negative mode differential metabolites with fold change values > 5. Additionally, Tyr Trp Trp Met and Asp His Lys expression was significantly reduced. Therefore, further investigation into the role of amino acids in disease will provide novel insights into potential links between BA and CS.

The lack of noninvasive markers renders the availability and reliability of markers for BA invaluable. The continuous exploration of novel biomarkers is anticipated to revolutionize the diagnosis of BA. Zahm et al.^21^ employed miRNAs to evaluate the discriminatory potential of serum miRNAS in distinguishing BA from other forms of neonatal hyperbilirubinemia. They observed a significantly higher expression of the miR-200b/429 cluster in the serum of BA patients compared to infants with non-BA cholestatic diseases, providing evidence for an elevated circulating level of the mR-200b/429 cluster as a promising diagnostic biomarker with clinical relevance. Furthermore, a recent study investigating serum matrix metalloproteinase (MMP)-7 levels revealed a significant elevation in children with BA compared to those without the condition (*P* < 0.0001). Notably, a serum MMP-7 level exceeding 1.43 ng/mL demonstrated an 88% diagnostic accuracy for predicting BA in children presenting with CS, suggesting its potential as a non-invasive biomarker^22^. In addition, several studies have demonstrated that IL-33, GGT, total protein, alkaline phosphatase, total bilirubin, and direct bilirubin can also serve as diagnostic markers for BA^23^. In this study, we also investigated the potential of developing a diagnostic model based on plasma metabolomics, yielding promising outcomes. In conclusion, for cases of unknown etiology in CS, it is crucial to have a rapid and accurate diagnosis and potentially avoid liver biopsy through an effective diagnostic model. Therefore, the development of our diagnostic model is anticipated to contribute to intervention in children with BA and improve prognosis.

Based on our findings, we have identified several aberrant metabolic pathways in BA, including sphingolipid metabolism. Sphingolipids serve as integral components of cellular membranes, playing crucial roles in the regulation of gene expression and cell signal transduction processes such as cell growth and apoptosis. Moreover, they act as key modulators of liver homeostasis, hepatic regeneration, and indicators for liver injury^24,25^. The lipid ceramide, derived from sphingolipid, functions as a signaling molecule in cellular differentiation and programmed cell death, playing a crucial role in the metabolism of sphingolipids^26^. Sphingolipids can also function as regulators in liver regeneration. Current studies have demonstrated that sphingolipid regulation can enhance cancer cell apoptosis in vitro and impede tumor growth in vivo; however, the mechanism of autophagy induced by sphingolipids remains unclear^27^. Our findings indicate that there is an aberration in sphingolipid metabolism in BA; however, further investigation is required to elucidate the precise underlying mechanism.

In the construction of the diagnostic model, we carefully selected two phosphocholines (PC) and one biliverdin. Bile PC plays a crucial role in safeguarding the cellular integrity of the bile duct lining against the cytotoxic effects induced by high concentrations of bile acids^28^. The reduction in PC availability or alteration of membrane phospholipid ratios leads to a decrease in bile acid secretion and the accumulation of bile acids in plasma and hepatocytes. This, in turn, may activate hepatic stellate cells and ultimately result in hepatic fibrosis^29^. Additionally, it has been demonstrated that impaired PC synthesis induces cholestasis in mice fed a high-fat diet, thus monitoring PC levels can aid in diagnosis. Moreover, we employed biliverdin to construct a diagnostic model. Under physiological conditions, the concentration of biliverdin in serum and bile is negligible compared to bilirubin^30^. Cholestasis in children is not characterized by a significant increase in serum bilirubin concentration, but rather by pronounced hyperbilirubinemia, which manifests as the characteristic yellow discoloration of the skin seen in jaundice. However, bilirubin accumulation has been observed in cases involving biliary obstruction, malnutrition, or severe liver damage^31^. The relationship between PC, biliverdin and BA, cholestasis is intricate, thus making them valuable diagnostic markers for both diseases.

## Conclusion

In this study, a total of 185 clinical plasma samples were analyzed, leading to the identification of 327 differential metabolites. The metabolomic characteristics of patients with BA and CS were elucidated. Furthermore, enrichment analysis revealed dysregulation of 10 metabolic pathways, including lysine degradation and fatty acid biosynthesis. Subsequently, a diagnostic model for BA was developed based on the combination of LysoPC(18:2(9Z,12Z)), PC(22:5(7Z,10Z,13Z,16Z,19Z)/14:0), and Biliverdin-IX-α. The area under the receiver operating characteristic curve was determined to be 0.968 with a sensitivity of 92.3% and specificity of 98.4%, thereby confirming the diagnostic efficacy for diagnosis of BA using this model. In summary, comprehensive metabolomics analysis enabled us to discern metabolic differences between BA and CS patients from multiple perspectives while also facilitating the development of a robust diagnostic model.

### Supplementary Information

Supplementary Information.

## Data Availability

The datasets generated and/or analyzed during the current study are not publicly available but are available from the corresponding author on reasonable request.
