# Supplementary material for: Biliary atresia and cholestasis plasma non-targeted metabolomics unravels perturbed metabolic pathways and unveils a diagnostic model for biliary atresia
Source: Sci Rep. 2024 Jul 9;14:15796. doi: 10.1038/s41598-024-66893-2 (PMC11233669; doi:10.1038/s41598-024-66893-2)
Supplement: Supplementary file 1 — Supplementary Information. [file 41598_2024_66893_MOESM1_ESM.docx]

**Supplemental Material**

**Biliary atresia and cholestasis plasma non-targeted metabolomics unravels perturbed metabolic pathways and unveils a diagnostic model for biliary atresia**

Bang Du^1^, Kai Mu^2^, Meng Sun^3^, Zhidan Yu^1^, Lifeng Li^1^, Ligong Hou^4^, Qionglin Wang^3^, Jushan Sun^1,*^, Jinhua Chen^5*^, Xianwei Zhang^1,2,*^, Wancun Zhang^1,3,4,*^

^1^Health Commission of Henan Province Key Laboratory for Precision Diagnosis and Treatment of Pediatric Tumor, Children’s Hospital Affiliated to Zhengzhou University, Zhengzhou, 450018, China

^2^Henan Key Laboratory of Rare Diseases, Endocrinology and Metabolism Center, The First Affiliated Hospital, and College of Clinical Medicine of Henan University of Science and Technology, Luoyang, 471003, China

^3^Henan Key Laboratory of Children’s Genetics and Metabolic Diseases, Children’s Hospital Affiliated to Zhengzhou University, Zhengzhou, 450018, China

^4^Henan International Joint Laboratory for Prevention and Treatment of Pediatric Disease, Children’s Hospital Affiliated to Zhengzhou University, Zhengzhou, 450018, China

^5^Department of Pharmacy, Affiliated Cancer Hospital of Zhengzhou University, Henan Cancer

Hospital, Zhengzhou 450008, China

* Corresponding author E-mail addresses: zhangwancun@126.com (Wancun Zhang); zhangxw956658@126.com (Xianwei Zhang); cjh070310216@163.com (Jinhua Chen); zzetyysjs@163.com (Jushan Sun)

Tel: + 86-373-63866536; Fax: +86-373-63866536;

^#^ These authors contributed equally to this work.

**Table S1** **Baseline Characteristics of BA and CS in metabolomics**

|  | BA | CS | NC | *P* Value |
| --- | --- | --- | --- | --- |
| Number | 90 | 48 | 47 | / |
| Age (month) | 1.70±0.92 | 1.90±1.10 | 3.88±3.12 | 0.195 |
| Male (%) | 53 | 61 | 38 | 0.355 |
| TBA (µmol/L) | 91.80±143.08 | 70.0±113.40 | / | 0.295 |
| TBIL (µmol/L) | 17.00±100.40 | 72.60±105.60 | / | 0.405 |
| DBIL (µmol/L) | 11.25±100.78 | 60.60±86.90 | / | 0.679 |
| ALT (U/L) | 96.05±118.50 | 105.00±84.90 | / | 0.359 |
| AST (U/L) | 81.80±144.70 | 81.60±93.90 | / | 0.452 |
| GGT (U/L) | 265.05±735.13 | 103.80±147.70 | / | ＜0.01 |

Note: *P* is the statistical test result between BA and CS.


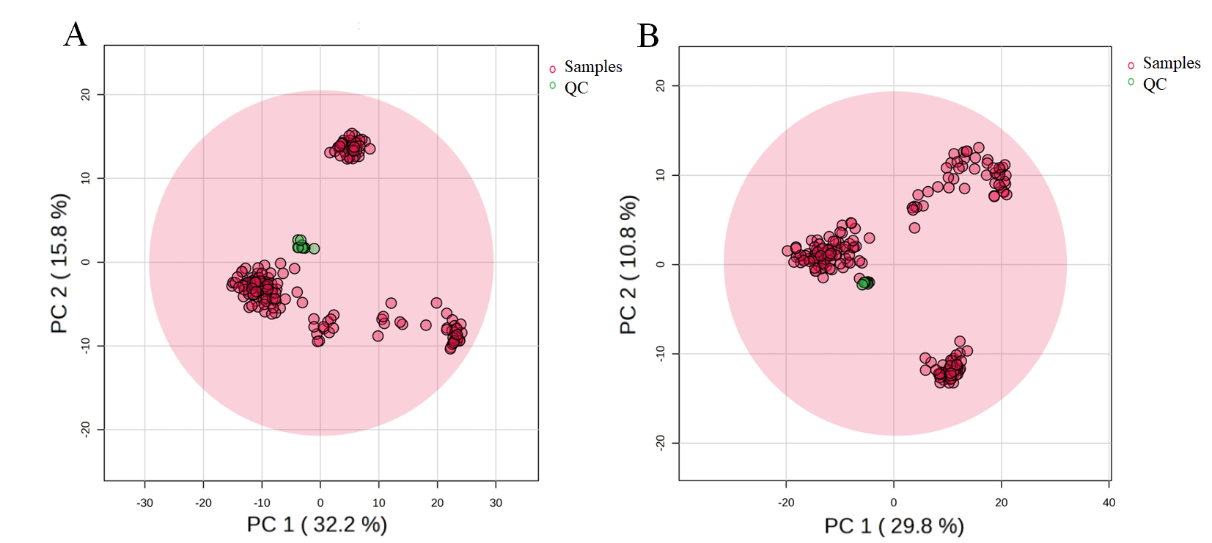


**Figure S1** The PCA plot between QC and samples. QC samples are closely clustered in (A) positive mode and (B) negative mode.


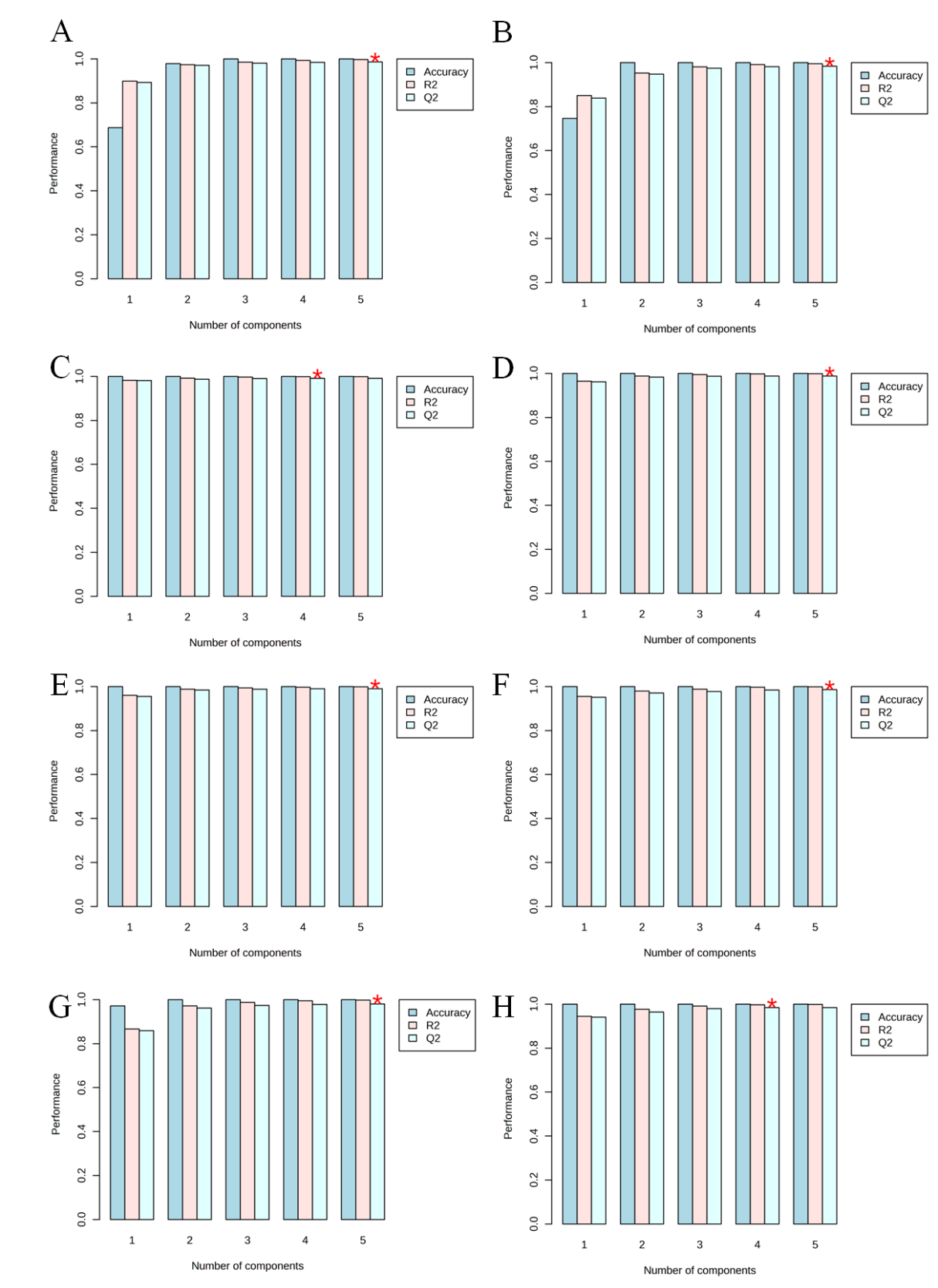


**Figure S2** The cross validation scores plot of the PLS-DA between BA and NC in positive (A) and negative modes (B), CS and NC in positive (C) and negative modes (D), BA and CS in positive (E) and negative modes (F).


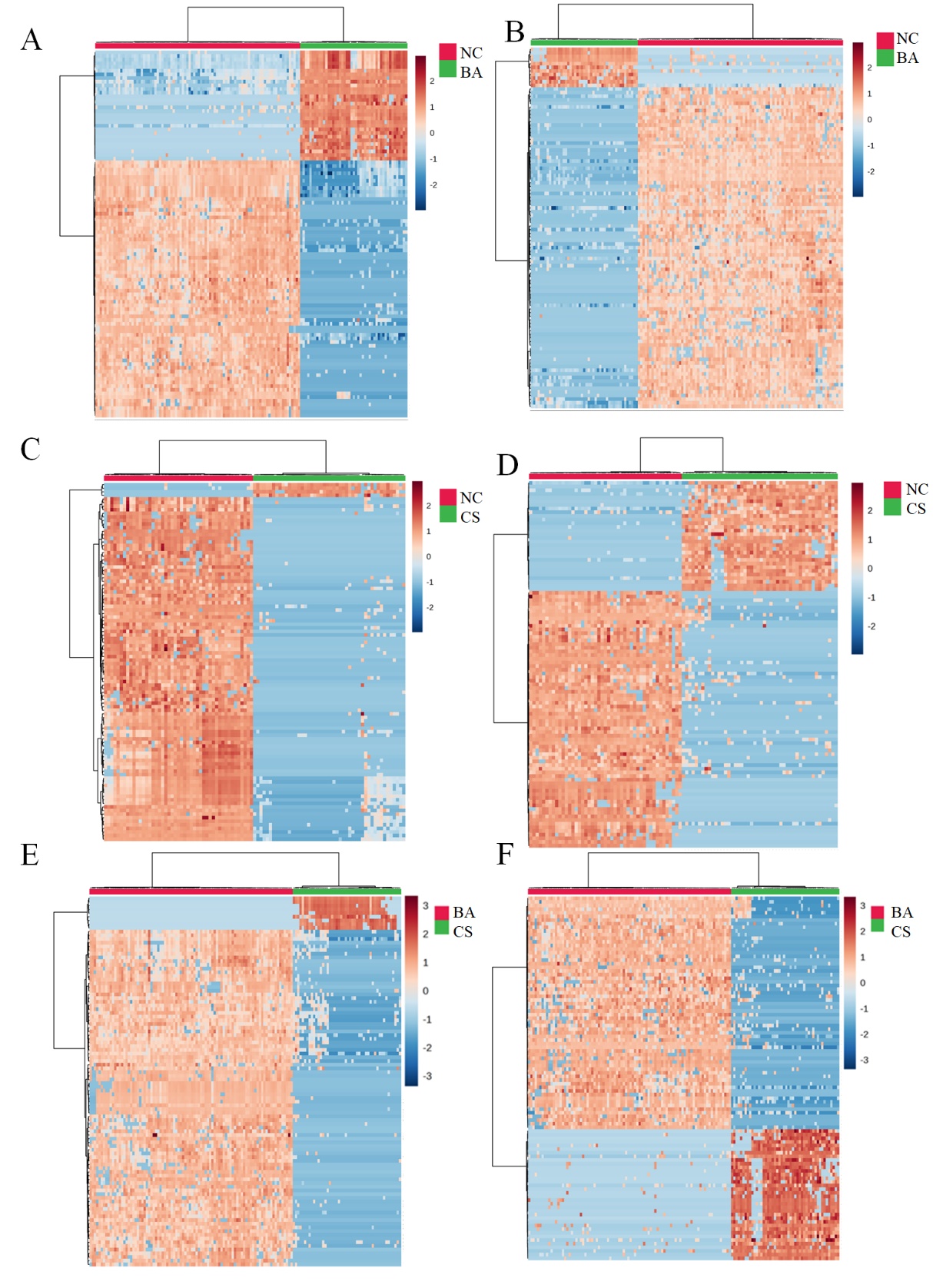


**Figure S3** The heatmaps of metabolite of BA *vs.* NC in positive mode (A) and negative mode (B), CS *vs.* NC in positive mode (C) and negative mode (D), BA *vs.* CS in positive mode (E) and negative mode (F).

**Table S2 Differentially expressed metabolites between BA and NC detected by positive ion mode acquisition**

| **NO.** | | **Metabolites** | **VIP**  **Value** | **Fold**  **Change** | ***P* Value** | **Regulation** | |
| --- | --- | --- | --- | --- | --- | --- | --- |
| 1 | Polyoxin B | | 3.7349 | 2110.8 | 4.25E-52 | | Up |
| 2 | Cefoperazone | | 3.6218 | 1691.5 | 7.99E-52 | | Up |
| 3 | alpha-Peroxyachifolide | | 3.0816 | 696.64 | 7.72E-53 | | Up |
| 4 | Pro Lys Pro Val | | 2.9218 | 81.78 | 3.91E-60 | | Up |
| 5 | C17 Sphinganine | | 2.6205 | 14.519 | 1.15E-53 | | Up |
| 6 | Glaucasterol | | 2.6074 | 87.014 | 3.46E-73 | | Up |
| 7 | Stigmatellin Y | | 2.586 | 0.0104 | 1.17E-66 | | Down |
| 8 | Methyl acetyl ricinoleate | | 2.2575 | 57.289 | 8.32E-28 | | Up |
| 9 | Cys His Arg Trp | | 2.0878 | 43.044 | 1.58E-57 | | Up |
| 10 | N-Acetylpyrrolidine | | 2.03 | 0.0248 | 1.09E-30 | | Down |
| 11 | Austalide B | | 2.0222 | 1403.5 | 2.73E-10 | | Up |
| 12 | (3Z)-Phytochromobilin | | 2.011 | 29.194 | 1.42E-47 | | Up |
| 13 | 5-Hydroxypropafenone | | 1.9445 | 45.114 | 2.18E-78 | | Up |
| 14 | Eicosanoyl-EA | | 1.9278 | 0.0246 | 1.33E-42 | | Down |
| 15 | Stearamide | | 1.9009 | 0.0358 | 3.82E-60 | | Down |
| 16 | Avocadene 4-acetate | | 1.8851 | 78.264 | 3.85E-31 | | Up |
| 17 | 2,2-Dimethyloxirane | | 1.879 | 57.087 | 9.86E-43 | | Up |
| 18 | Aquifoliunine EIII | | 1.8352 | 52.322 | 3.56E-37 | | Up |
| 19 | Montanol | | 1.831 | 2.7714 | 2.74E-21 | | Up |
| 20 | 22-Hydroxyvitamin D3 | | 1.7924 | 18.298 | 1.58E-44 | | Up |
| 21 | 13,14-dihydroxy-docosanoic acid | | 1.7922 | 0.0241 | 2.19E-47 | | Down |
| 22 | Mannosyl-1beta-phosphomycoketide C30 | | 1.7277 | 30.186 | 2.42E-26 | | Up |
| 23 | 5,5-Diisopropyl-2,2'-dimethylbiphenyl-3,3',4,4'-tetrone | | 1.6888 | 28.137 | 3.55E-68 | | Up |
| 24 | Pancratistatin | | 1.5955 | 259.08 | 2.15E-10 | | Up |
| 25 | Hydrolysis product of bussein | | 1.5808 | 27.62 | 1.88E-25 | | Up |
| 26 | Acetyl tributyl citrate | | 1.5649 | 7.4534 | 3.73E-20 | | Up |
| 27 | Halaminol A | | 1.5179 | 0.0568 | 4.62E-83 | | Down |
| 28 | PC(22:6(4Z,7Z,10Z,13Z,16Z,19Z)/16:0) | | 1.4926 | 56.859 | 1.09E-07 | | Up |
| 29 | Ambronide | | 1.4836 | 15.868 | 3.19E-24 | | Up |
| 30 | 5-Methyl-2,5-di-1-pyrrolidinyl-2-cyclopenten-1-one | | 1.481 | 11.942 | 5.87E-19 | | Up |
| 31 | Malyngamide H | | 1.4716 | 10.346 | 2.00E-41 | | Up |
| 32 | dodecanamide | | 1.4623 | 0.0457 | 8.66E-55 | | Down |
| 33 | DG(14:1(9Z)/22:3(10Z,13Z,16Z)/0:0)[iso2] | | 1.4442 | 10.428 | 7.80E-20 | | Up |
| 34 | Arg Arg Lys Ala | | 1.4257 | 0.1123 | 5.46E-33 | | Down |
| 35 | (R)-2-Hydroxysterculic acid | | 1.4043 | 28.439 | 1.10E-24 | | Up |
| 36 | Polyoxyethylene 40 monostearate | | 1.394 | 0.0507 | 3.42E-44 | | Down |
| 37 | 3-Methoxy-4-hydroxyphenylethylene glycol | | 1.3824 | 37.473 | 1.71E-27 | | Up |
| 38 | 5-Butyl-8-methyloctahydroindolizin-8-ol | | 1.369 | 0.0635 | 1.42E-54 | | Down |
| 39 | PS(18:0/0:0) | | 1.3428 | 0.0734 | 1.84E-34 | | Down |
| 40 | 9-methoxy-pentadecanoic acid | | 1.3378 | 0.0662 | 4.06E-28 | | Down |
| 41 | Bisindolylmaleimide I | | 1.299 | 13.535 | 1.80E-56 | | Up |
| 42 | N-docosahexaenoyl GABA | | 1.2975 | 8.2035 | 5.34E-45 | | Up |
| 43 | Glu Arg Leu | | 1.2945 | 10.579 | 8.78E-29 | | Up |
| 44 | Cucurbitacin S | | 1.2939 | 0.2748 | 7.57E-20 | | Down |
| 45 | Phytosphingosine | | 1.289 | 0.0586 | 3.84E-43 | | Down |
| 46 | Glycidyl oleate | | 1.2757 | 13.204 | 1.58E-27 | | Up |
| 47 | Biliverdin-IX-α | | 1.2616 | 12.573 | 1.94E-37 | | Up |
| 48 | PC(O-2:0/2:0) | | 1.2599 | 32.315 | 9.08E-28 | | Up |
| 49 | Enigmol | | 1.2583 | 0.0683 | 2.53E-48 | | Down |
| 50 | 2-bromo-octadecanoic acid | | 1.2304 | 15.462 | 9.50E-23 | | Up |
| 51 | Bisibuthiamine | | 1.2189 | 12.266 | 1.00E-49 | | Up |
| 52 | C16 Sphinganine | | 1.2131 | 0.0775 | 5.01E-49 | | Down |
| 53 | Geijerone | | 1.2089 | 8.1736 | 7.77E-18 | | Up |
| 54 | Tecostanine | | 1.2027 | 0.0797 | 7.18E-50 | | Down |
| 55 | MG(22:6(4Z,7Z,10Z,13Z,16Z,19Z)/0:0/0:0) | | 1.1874 | 14.743 | 1.29E-18 | | Up |
| 56 | Anandamide (20:l, n-9) | | 1.1819 | 0.1203 | 2.56E-28 | | Down |
| 57 | 13-methoxy-heneicosanoic acid | | 1.1777 | 0.1084 | 8.81E-34 | | Down |
| 58 | 10,20-Dihydroxyeicosanoic acid | | 1.1441 | 0.0747 | 1.42E-32 | | Down |
| 59 | Ascorbic acid | | 1.1384 | 16.639 | 5.20E-20 | | Up |
| 60 | Methyl (R)-9-hydroxy-10-undecene-5,7-diynoate glucoside | | 1.1332 | 14.132 | 8.72E-28 | | Up |
| 61 | 6-Oxocativic acid | | 1.1297 | 14.799 | 2.34E-18 | | Up |
| 62 | 1-(4-Amino-2-methylpyrimid-5-ylmethyl)-3-(beta-hydroxyethyl)-2-methylpyridinium | | 1.1189 | 22.42 | 3.13E-18 | | Up |
| 63 | Hexadecan-3-one | | 1.1095 | 0.0895 | 2.06E-43 | | Down |
| 64 | Val Arg Pro Lys | | 1.0959 | 22.956 | 8.05E-13 | | Up |
| 65 | PC(22:4(7Z,10Z,13Z,16Z)/12:0) | | 1.0894 | 13.786 | 1.22E-14 | | Up |
| 66 | 4-(2,6,6-Trimethylcyclohexa-1,3-dienyl)but-3-en-2-one | | 1.0862 | 12.547 | 3.97E-23 | | Up |
| 67 | Phe Phe Gln Trp | | 1.07 | 6.2159 | 3.18E-41 | | Up |
| 68 | 1,3-Octadiene | | 1.0651 | 0.0839 | 4.75E-28 | | Down |
| 69 | N-palmitoyl threonine | | 1.0373 | 0.2431 | 1.65E-18 | | Down |
| 70 | 4-Vinylcyclohexene | | 1.0268 | 4.8011 | 2.99E-10 | | Up |
| 71 | Glu Glu Glu Asp | | 1.0135 | 0.2190 | 6.22E-15 | | Down |
| 72 | PC(22:5(7Z,10Z,13Z,16Z,19Z)/16:0) | | 1.0058 | 22.072 | 3.18E-07 | | Up |

**Table S3 Differentially expressed metabolites between BA and NC detected by negative ion mode acquisition**

| **NO.** | | **Metabolites** | **VIP**  **Value** | **Fold**  **Change** | ***P* Value** | **Regulation** | |
| --- | --- | --- | --- | --- | --- | --- | --- |
| 1 | Dopamine 3-O-sulfate | | 3.0355 | 1264.9 | 2.69E-31 | | Up |
| 2 | Arg Cys Glu Arg | | 2.726 | 158.92 | 8.18E-63 | | Up |
| 3 | Scillipheosidin 3-[glucosyl-(1-2)-rhamnoside] | | 1.9544 | 1668.1 | 1.05E-10 | | Up |
| 4 | 3,9-Dimethyluric acid | | 1.9408 | 88.493 | 6.84E-22 | | Up |
| 5 | Phe Asp Asp Asp | | 1.8847 | 41.155 | 2.02E-31 | | Up |
| 6 | Ethyl vanillin isobutyrate | | 1.794 | 0.0856 | 1.63E-31 | | Down |
| 7 | (4-tert-Butyl-phenoxy)-acetic acid | | 1.7609 | 65.805 | 6.36E-32 | | Up |
| 8 | Trp Ser Pro Pro | | 1.7265 | 25.322 | 1.79E-17 | | Up |
| 9 | Difenoxuron | | 1.7092 | 24.695 | 4.21E-34 | | Up |
| 10 | Leu Met Trp Met | | 1.7034 | 26.424 | 3.29E-17 | | Up |
| 11 | N-acetyl-S-geranylgeranyl-L-Cysteine | | 1.6778 | 48.604 | 1.51E-18 | | Up |
| 12 | 2-Methyl-3-(2-pentenyl)-2-cyclopenten-1-one | | 1.6333 | 43.133 | 4.50E-29 | | Up |
| 13 | 1-Phosphatidyl-D-myo-inositol | | 1.6333 | 0.0246 | 1.58E-24 | | Down |
| 14 | 7beta,12beta-Dihydroxy-5alpha-cholan-24-oic Acid | | 1.6025 | 0.15266 | 6.68E-19 | | Down |
| 15 | Anisole | | 1.5443 | 0.0108 | 6.46E-16 | | Down |
| 16 | PA(O-16:0/12:0) | | 1.5132 | 35.464 | 3.54E-15 | | Up |
| 17 | Ptilosteroid B | | 1.4994 | 6.2625 | 6.42E-24 | | Up |
| 18 | Gln Glu Asp Cys | | 1.4763 | 121.72 | 8.57E-12 | | Up |
| 19 | Guvacoline | | 1.4711 | 29.548 | 4.17E-32 | | Up |
| 20 | Br-Honaucin A | | 1.4197 | 281.33 | 2.41E-07 | | Up |
| 21 | His Gly His Trp | | 1.3892 | 26.387 | 2.34E-28 | | Up |
| 22 | Alpha-N-Phenylacetyl-L-glutamine | | 1.3534 | 0.0961 | 3.01E-16 | | Down |
| 23 | Pyrimidine | | 1.3478 | 25.746 | 6.06E-24 | | Up |
| 24 | Indoxylsulfuric acid | | 1.3407 | 0.2565 | 5.73E-13 | | Down |
| 25 | 2,3-Dihydro-5,5',7,7'-tetrahydroxy-2-(4-hydroxyphenyl)[3,8'-bi-4H-1-benzopyran]-4,4'-dione | | 1.3214 | 0.0916 | 2.47E-19 | | Down |
| 26 | Acetylaminodantrolene | | 1.2756 | 18.88 | 2.78E-25 | | Up |
| 27 | N-depyridomethyl-Indinavir | | 1.241 | 14.697 | 1.71E-22 | | Up |
| 28 | Octadecanedioic acid | | 1.2299 | 11.865 | 2.09E-13 | | Up |
| 29 | Semilepidinoside A | | 1.2113 | 0.0616 | 1.40E-32 | | Down |
| 30 | 2-Furylmercury chloride | | 1.1858 | 0.0854 | 3.79E-36 | | Down |
| 31 | Metominostrobin | | 1.1639 | 12.132 | 7.89E-14 | | Up |
| 32 | DMABA-d6 NHS ester | | 1.1585 | 0.1501 | 9.59E-25 | | Down |
| 33 | Glucosereductone | | 1.1159 | 0.3050 | 1.82E-11 | | Down |
| 34 | 8-Methyl-5-propyloctahydroindolizin-8-ol | | 1.1063 | 0.2037 | 5.23E-19 | | Down |
| 35 | Ronidazole | | 1.0977 | 0.0567 | 5.49E-10 | | Down |
| 36 | Stilbamidine | | 1.0738 | 0.0802 | 3.62E-39 | | Down |
| 37 | Oxalosuccinic acid | | 1.069 | 0.0581 | 1.44E-12 | | Down |
| 38 | Thr Met Glu Asp | | 1.0525 | 4.1376 | 3.40E-16 | | Up |
| 39 | Psoromic acid | | 1.0315 | 0.0291 | 9.63E-16 | | Down |
| 40 | DL-2-hydroxy stearic acid | | 1.0083 | 6.6256 | 4.19E-26 | | Up |

**Table S4 Differentially expressed metabolites between CS and NC detected by positive ion mode acquisition**

| **NO.** | | **Metabolites** | **VIP**  **Value** | **Fold**  **Change** | ***P* Value** | **Regulation** | |
| --- | --- | --- | --- | --- | --- | --- | --- |
| 1 | Stearamide | | 2.9102 | 101.49 | 5.37E-42 | | Up |
| 2 | LysoPC(18:2(9Z,12Z)) | | 2.5191 | 2.7788 | 7.03E-13 | | Up |
| 3 | PE(19:0/0:0) | | 2.5035 | 3.377 | 4.53E-14 | | Up |
| 4 | PE(22:6(4Z,7Z,10Z,13Z,16Z,19Z)/0:0) | | 2.4763 | 3.7737 | 3.42E-27 | | Up |
| 5 | N-Hexadecanoylpyrrolidine | | 2.4437 | 70.126 | 7.72E-45 | | Up |
| 6 | N-Acetylpyrrolidine | | 2.4147 | 76.528 | 7.76E-33 | | Up |
| 7 | Palmitic amide | | 2.4034 | 70.958 | 4.30E-48 | | Up |
| 8 | Polyoxyethylene 40 monostearate | | 2.3981 | 74.965 | 7.30E-38 | | Up |
| 9 | 3,4-Epoxy-6,9-octadecadiene | | 2.2844 | 18.034 | 9.50E-27 | | Up |
| 10 | Enigmol | | 2.1505 | 50.867 | 9.52E-38 | | Up |
| 11 | Pentyl heptanoate | | 2.1351 | 61.721 | 1.07E-39 | | Up |
| 12 | Docosanamide | | 2.1127 | 42.249 | 2.95E-48 | | Up |
| 13 | 9-methoxy-pentadecanoic acid | | 2.1007 | 68.815 | 1.93E-39 | | Up |
| 14 | MG(0:0/18:0/0:0) | | 2.0153 | 10.469 | 4.20E-22 | | Up |
| 15 | 3-Hydroxyethylchlorophyllide a | | 1.9718 | 33.392 | 4.22E-41 | | Up |
| 16 | 13-methoxy-heneicosanoic acid | | 1.9678 | 34.418 | 2.23E-41 | | Up |
| 17 | Phytosphingosine | | 1.908 | 38.011 | 1.17E-30 | | Up |
| 18 | Trp Gln Phe Phe | | 1.8841 | 44.802 | 9.25E-55 | | Up |
| 19 | DG(15:0/19:0/0:0)[iso2] | | 1.8715 | 30.359 | 1.63E-15 | | Up |
| 20 | Eicosanoyl-EA | | 1.8632 | 33.528 | 3.09E-32 | | Up |
| 21 | C16 Sphinganine | | 1.857 | 32.385 | 4.50E-35 | | Up |
| 22 | PC(20:2(11Z,14Z)/0:0) | | 1.8326 | 9.1535 | 1.63E-33 | | Up |
| 23 | PE(20:0/0:0) | | 1.8171 | 7.2524 | 3.48E-21 | | Up |
| 24 | MG(16:0/0:0/0:0)[rac] | | 1.7466 | 5.0144 | 1.98E-16 | | Up |
| 25 | SM(d18:2/14:0) | | 1.7434 | 16.098 | 2.17E-45 | | Up |
| 26 | 10,20-Dihydroxyeicosanoic acid | | 1.7187 | 29.318 | 1.38E-27 | | Up |
| 27 | 6,7-Epoxy-9Z-octadecene | | 1.7154 | 11.208 | 7.82E-25 | | Up |
| 28 | Diphenylcarbazide | | 1.7114 | 7.7341 | 1.32E-23 | | Up |
| 29 | LysoPC(14:0) | | 1.7005 | 3.519 | 9.69E-17 | | Up |
| 30 | Tyr Trp Phe Ala | | 1.6491 | 16.522 | 2.60E-43 | | Up |
| 31 | 6α-Hydroxy-3-oxo-5β-cholan-24-oic Acid | | 1.6152 | 2.0972 | 1.29E-09 | | Up |
| 32 | Bargustanine | | 1.6048 | 7.8158 | 3.52E-27 | | Up |
| 33 | hellebrin | | 1.6037 | 54.197 | 3.75E-33 | | Up |
| 34 | Saccharopine | | 1.5934 | 9.0173 | 1.78E-27 | | Up |
| 35 | 13E-Docosenamide | | 1.59 | 11.179 | 4.02E-28 | | Up |
| 36 | LysoPC(22:6(4Z,7Z,10Z,13Z,16Z,19Z)) | | 1.59 | 2.3901 | 2.71E-12 | | Up |
| 37 | N-(2,5-Dihydroxyphenyl)pyridinium | | 1.5856 | 3.4473 | 4.23E-12 | | Up |
| 38 | Halaminol A | | 1.5449 | 21.656 | 1.68E-57 | | Up |
| 39 | PC(O-16:0/0:0)[U] | | 1.5242 | 5.458 | 8.53E-19 | | Up |
| 40 | 8-Methyl-5-propyloctahydroindolizin-8-ol | | 1.5067 | 14.02 | 3.47E-28 | | Up |
| 41 | Linoleamide | | 1.5031 | 5.3153 | 4.88E-27 | | Up |
| 42 | Phe Phe Gln Arg | | 1.4934 | 9.2602 | 1.19E-23 | | Up |
| 43 | PC(22:4(7Z,10Z,13Z,16Z)/0:0) | | 1.4916 | 15.081 | 7.52E-42 | | Up |
| 44 | 4-(Trimethylammonio)but-2-enoate | | 1.4873 | 38.774 | 1.19E-18 | | Up |
| 45 | 13,14-dihydroxy-docosanoic acid | | 1.4378 | 28.494 | 3.58E-30 | | Up |
| 46 | LysoPC(22:5(7Z,10Z,13Z,16Z,19Z)) | | 1.4311 | 2.5165 | 3.37E-16 | | Up |
| 47 | 3,4-Dimethylstyrene | | 1.4222 | 10.664 | 7.71E-22 | | Up |
| 48 | SM(d16:1/16:0) | | 1.4159 | 25.729 | 3.62E-29 | | Up |
| 49 | Linoleyl carnitine | | 1.3947 | 4.8245 | 1.36E-19 | | Up |
| 50 | Phytanal | | 1.3915 | 17.51 | 5.11E-60 | | Up |
| 51 | 1-Hexadecen-3-one | | 1.3806 | 3.8528 | 1.38E-14 | | Up |
| 52 | PE(P-16:0/0:0) | | 1.3747 | 5.1793 | 8.93E-21 | | Up |
| 53 | Polyoxyethylene (600) mono- ricinoleate | | 1.3613 | 8.2652 | 8.97E-28 | | Up |
| 54 | PC(15:1(9Z)/0:0) | | 1.3601 | 2.0793 | 4.43E-13 | | Up |
| 55 | Nonoxynol-9 | | 1.3586 | 11.546 | 3.17E-36 | | Up |
| 56 | dodecanamide | | 1.3522 | 21.874 | 6.70E-34 | | Up |
| 57 | Edetate | | 1.3469 | 4.7745 | 2.60E-13 | | Up |
| 58 | Phosphodimethylethanolamine | | 1.3426 | 4.7637 | 1.26E-15 | | Up |
| 59 | Biliverdin-IX-α | | 1.3331 | 2.3835 | 5.87E-15 | | Up |
| 60 | 1,3-Octadiene | | 1.2931 | 29.086 | 6.82E-18 | | Up |
| 61 | Tetradecyl isobutyrate | | 1.2705 | 6.2082 | 2.74E-26 | | Up |
| 62 | (±)-Octanoylcarnitine | | 1.2672 | 3.9335 | 8.93E-17 | | Up |
| 63 | (2R,6R,7S,8S)-7-Ethyl-2-propyl-1-azaspiro[5.5]undecan-8-ol | | 1.2659 | 3.8504 | 1.32E-26 | | Up |
| 64 | 2-Methyl-3'-hydroxyphenylpropionic acid | | 1.2588 | 13.275 | 1.98E-18 | | Up |
| 65 | Threoninyl-Aspartate | | 1.2515 | 12.332 | 2.33E-14 | | Up |
| 66 | 5-Butyl-8-methyloctahydroindolizin-8-ol | | 1.2338 | 15.964 | 4.44E-37 | | Up |
| 67 | 9,10-dihydroxy-hexadecanoic acid | | 1.2211 | 14.83 | 6.18E-19 | | Up |
| 68 | N-Oleoyl-L-Serine | | 1.2206 | 4.196 | 1.99E-16 | | Up |
| 69 | 2,6-nonadienal | | 1.189 | 4.8002 | 1.54E-13 | | Up |
| 70 | PC(18:3(6Z,9Z,12Z)/0:0) | | 1.1574 | 3.0287 | 3.01E-09 | | Up |
| 71 | Xestoaminol C | | 1.1315 | 4.9048 | 2.86E-17 | | Up |
| 72 | Tecostanine | | 1.1215 | 13.207 | 3.40E-32 | | Up |
| 73 | 3-cis-Hydroxy-b,e-Caroten-3'-one | | 1.1162 | 8.6293 | 1.32E-30 | | Up |
| 74 | (2E,4E)-2,4-Dodecadienal | | 1.1105 | 4.925 | 1.10E-11 | | Up |
| 75 | Isoquinoline N-oxide | | 1.1078 | 3.7884 | 3.30E-12 | | Up |
| 76 | PC(17:2(9Z,12Z)/12:0) | | 1.1028 | 17.635 | 5.22E-19 | | Up |
| 77 | C-8 Ceramide-1-phosphate | | 1.0997 | 6.5913 | 9.98E-24 | | Up |
| 78 | Pahutoxin | | 1.0895 | 2.3876 | 6.80E-14 | | Up |
| 79 | Isopalmitic acid | | 1.0843 | 2.7207 | 2.30E-15 | | Up |
| 80 | (4'R,6R,6'S,7S,8R)-6'-(2-Hydroxypentan-2-yl)-4',8-dimethyldecahydro-5H-spiro[indolizine-6,2'-pyran]-7,8-diol | | 1.0799 | 3.2013 | 4.36E-13 | | Up |
| 81 | PC(O-16:2(9E,10E)/0:0)[U] | | 1.0758 | 5.5184 | 1.90E-25 | | Up |
| 82 | Tyr Trp Arg Ala | | 1.0652 | 6.599 | 1.72E-21 | | Up |
| 83 | Anandamide (20:l, n-9) | | 1.0593 | 8.9728 | 3.43E-26 | | Up |
| 84 | LysoPC(18:1(11Z)) | | 1.0584 | 2.7923 | 1.94E-15 | | Up |
| 85 | C14:1n-10 | | 1.0524 | 3.908 | 2.49E-16 | | Up |
| 86 | Clausarinol | | 1.0479 | 3.6407 | 9.71E-14 | | Up |
| 87 | Thr Ala Arg | | 1.0362 | 5.2967 | 2.31E-16 | | Up |
| 88 | DG(14:1(9Z)/22:3(10Z,13Z,16Z)/0:0)[iso2] | | 1.0305 | 0.1238 | 2.15E-09 | | Down |
| 89 | L-Hexanoylcarnitine n-butyl ester | | 1.023 | 3.7192 | 1.15E-16 | | Up |
| 90 | Pipericine | | 1.0116 | 8.1498 | 1.64E-18 | | Up |
| 91 | LysoPC(0:0/18:0) | | 1.0084 | 2.9515 | 4.49E-13 | | Up |
| 92 | N-palmitoyl-phosphoethanolamine | | 1.0058 | 3.6926 | 7.56E-17 | | Up |

**Table S5 Differentially expressed metabolites between CS and NC detected by negative ion mode acquisition**

| **NO.** | | **Metabolites** | **VIP**  **Value** | **Fold**  **Change** | ***P* Value** | **Regulation** | |
| --- | --- | --- | --- | --- | --- | --- | --- |
| 1 | Leu Met Trp Met | | 2.0441 | 36.905 | 6.19E-27 | | Up |
| 2 | 2,3-Dihydro-5,5',7,7'-tetrahydroxy-2-(4-hydroxyphenyl)[3,8'-bi-4H-1-benzopyran]-4,4'-dione | | 1.8647 | 142.12 | 7.65E-26 | | Up |
| 3 | Thr Cys Glu Pro Ile | | 1.762 | 12.97 | 5.04E-27 | | Up |
| 4 | Ethyl vanillin isobutyrate | | 1.7094 | 15.537 | 7.61E-29 | | Up |
| 5 | Glucosereductone | | 1.7082 | 16.509 | 1.15E-30 | | Up |
| 6 | Asp His Lys | | 1.5916 | 0.0123 | 3.90E-15 | | Down |
| 7 | Aflatoxin GM1 | | 1.5509 | 93.36 | 7.52E-19 | | Up |
| 8 | Edetate | | 1.4051 | 6.3021 | 5.74E-13 | | Up |
| 9 | TyrMe-TyrMe-OH | | 1.3889 | 2.5882 | 7.24E-14 | | Up |
| 10 | Semilepidinoside A | | 1.3308 | 24.99 | 1.18E-21 | | Up |
| 11 | Anhydrocinnzeylanine | | 1.2778 | 12.504 | 3.21E-22 | | Up |
| 12 | Alpha-N-Phenylacetyl-L-glutamine | | 1.2513 | 14.658 | 2.25E-15 | | Up |
| 13 | Clovamide | | 1.2367 | 6.6278 | 5.85E-26 | | Up |
| 14 | Anisole | | 1.2277 | 60.46 | 4.42E-09 | | Up |
| 15 | Sodium glycocholate | | 1.2078 | 0.3451 | 1.68E-07 | | Down |
| 16 | Ronidazole | | 1.1833 | 52.628 | 8.52E-09 | | Up |
| 17 | Lucidenic acid N | | 1.1826 | 13.765 | 8.59E-26 | | Up |
| 18 | Celereoin | | 1.1812 | 20.221 | 9.23E-20 | | Up |
| 19 | 5,6-Dihydro-11-methoxyyangonin | | 1.1401 | 4.8264 | 2.11E-26 | | Up |
| 20 | 5-Acetylamino-6-formylamino-3-methyluracil | | 1.1238 | 6.351 | 6.16E-19 | | Up |
| 21 | Trp Trp Arg Met | | 1.0735 | 4.1433 | 1.17E-15 | | Up |
| 22 | 8-Methyl-5-propyloctahydroindolizin-8-ol | | 1.045 | 8.9079 | 2.22E-27 | | Up |
| 23 | Clovanediol diacetate | | 1.0382 | 2.8882 | 3.71E-13 | | Up |
| 24 | Trp Ser Pro Pro | | 1.0305 | 17.699 | 1.70E-14 | | Up |
| 25 | Phe Ala Arg | | 1.0029 | 4.499 | 4.38E-20 | | Up |

**Table S6 Differentially expressed metabolites between BA and CS detected by positive ion mode acquisition**

| **NO.** | | **Metabolites** | **VIP**  **Value** | **Fold**  **Change** | ***P* Value** | **Regulation** | |
| --- | --- | --- | --- | --- | --- | --- | --- |
| 1 | Cefoperazone | | 2.886 | 7.0851 | 4.13E-30 | | Up |
| 2 | LysoPC(18:2(9Z,12Z)) | | 2.5601 | 2.8636 | 4.79E-22 | | Up |
| 3 | PC(22:5(7Z,10Z,13Z,16Z,19Z)/14:0) | | 2.5332 | 106.35 | 2.25E-19 | | Up |
| 4 | Biliverdin-IX-α | | 2.5328 | 30.103 | 5.19E-47 | | Up |
| 5 | PE(22:6(4Z,7Z,10Z,13Z,16Z,19Z)/0:0) | | 2.1853 | 2.8843 | 1.54E-22 | | Up |
| 6 | Boviquinone 4 | | 2.0791 | 3.6236 | 5.68E-24 | | Up |
| 7 | Linoleyl carnitine | | 1.9051 | 5.5577 | 1.80E-26 | | Up |
| 8 | 3-(2-Heptenyloxy)-2-hydroxypropyl undecanoate | | 1.8544 | 3.6611 | 3.05E-15 | | Up |
| 9 | Methyl acetyl ricinoleate | | 1.815 | 2.1418 | 1.90E-15 | | Up |
| 10 | Acetyl tributyl citrate | | 1.6855 | 2.3442 | 4.48E-19 | | Up |
| 11 | 3,4-Epoxy-6,9-octadecadiene | | 1.679 | 4.5033 | 4.68E-22 | | Up |
| 12 | PC(O-16:0/0:0)[U] | | 1.6602 | 6.2743 | 6.18E-44 | | Up |
| 13 | 6α-Hydroxy-3-oxo-5β-cholan-24-oic Acid | | 1.6327 | 2.1941 | 3.24E-16 | | Up |
| 14 | 25-Methyl-21-tritriacontene-1,9,11-triol | | 1.6251 | 34.377 | 6.37E-28 | | Up |
| 15 | Aquifoliunine EIII | | 1.6032 | 24.997 | 1.24E-27 | | Up |
| 16 | PE(20:3(8Z,11Z,14Z)/0:0) | | 1.5797 | 8.2906 | 2.84E-40 | | Up |
| 17 | Trp Gln Phe Phe | | 1.5394 | 13.945 | 1.54E-28 | | Up |
| 18 | C18:3n-5,7,9 | | 1.5309 | 3.1975 | 4.84E-18 | | Up |
| 19 | PI(20:4(5Z,8Z,11Z,14Z)/0:0) | | 1.5162 | 8.6029 | 2.61E-46 | | Up |
| 20 | PS(O-18:0/0:0) | | 1.5088 | 2.2731 | 1.30E-21 | | Up |
| 21 | Cys His Arg Trp | | 1.5055 | 5.8324 | 3.13E-29 | | Up |
| 22 | Metipranolol | | 1.4854 | 4.5626 | 2.07E-16 | | Up |
| 23 | PC(O-2:0/2:0) | | 1.4217 | 38.732 | 1.37E-32 | | Up |
| 24 | Pirimicarb | | 1.4055 | 19.134 | 3.02E-16 | | Up |
| 25 | 5-Hydroxypropafenone | | 1.4037 | 6.6973 | 2.50E-35 | | Up |
| 26 | Austalide B | | 1.4027 | 5.3683 | 9.99E-06 | | Up |
| 27 | 9Z,12Z,15Z-Octadecatrienal | | 1.3786 | 2.546 | 8.61E-15 | | Up |
| 28 | MG(16:0/0:0/0:0)[rac] | | 1.3769 | 2.2182 | 3.46E-18 | | Up |
| 29 | N-(2,5-Dihydroxyphenyl) pyridinium | | 1.3706 | 2.7686 | 3.89E-21 | | Up |
| 30 | Diphenylcarbazide | | 1.3132 | 3.3181 | 8.63E-27 | | Up |
| 31 | PC(O-11:1(10E)/2:0) | | 1.3052 | 2.826 | 3.65E-26 | | Up |
| 32 | SM(d18:2/14:0) | | 1.2995 | 12.591 | 6.66E-49 | | Up |
| 33 | Pahutoxin | | 1.2979 | 4.2357 | 2.58E-24 | | Up |
| 34 | Isolinderanolide | | 1.2858 | 2.553 | 3.73E-18 | | Up |
| 35 | Tyr Met Cys Cys | | 1.2701 | 6.1661 | 1.25E-09 | | Up |
| 36 | Glycidyl oleate | | 1.2622 | 2.9686 | 1.35E-18 | | Up |
| 37 | Diisobutyl phthalate | | 1.2423 | 2.5371 | 8.47E-17 | | Up |
| 38 | 7,7',8,8'-Tetrahydrolycopene | | 1.2365 | 9.1955 | 4.85E-38 | | Up |
| 39 | Isopalmitic acid | | 1.2332 | 4.3828 | 9.73E-25 | | Up |
| 40 | Pro Gln Arg Trp | | 1.2275 | 9.5103 | 1.60E-28 | | Up |
| 41 | Pancratistatin | | 1.2203 | 10.505 | 4.57E-07 | | Up |
| 42 | Cys Asp Asp Glu | | 1.2072 | 19.803 | 1.44E-21 | | Up |
| 43 | 3-oxo-nonadecanoic acid | | 1.2017 | 2.7545 | 4.84E-22 | | Up |
| 44 | (±)-Octanoylcarnitine | | 1.1899 | 3.5195 | 2.79E-23 | | Up |
| 45 | N-Oleoyl-L-Serine | | 1.1875 | 3.5716 | 4.51E-17 | | Up |
| 46 | 6,7-Epoxy-9Z-octadecene | | 1.1815 | 3.8653 | 3.12E-24 | | Up |
| 47 | 12-Shogaol | | 1.1808 | 3.5314 | 2.55E-23 | | Up |
| 48 | 3,7-Dimethyl-2E,6E-decadien-1,10-dioic acid | | 1.1566 | 3.7504 | 4.30E-22 | | Up |
| 49 | C18:1n-3 | | 1.1508 | 2.6642 | 5.46E-12 | | Up |
| 50 | (3Z)-Phytochromobilin | | 1.1504 | 7.6268 | 1.99E-09 | | Up |
| 51 | 1-Hexadecen-3-one | | 1.1457 | 2.4611 | 3.96E-18 | | Up |
| 52 | 3-Deoxyvitamin D3 | | 1.1388 | 2.475 | 4.69E-12 | | Up |
| 53 | Oleamide | | 1.1369 | 2.4976 | 1.30E-12 | | Up |
| 54 | N-palmitoyl glutamic acid | | 1.1364 | 7.4337 | 2.08E-16 | | Up |
| 55 | Stearamide | | 1.1353 | 3.4578 | 1.52E-19 | | Up |
| 56 | PS(O-20:0/0:0) | | 1.1316 | 2.9293 | 8.22E-24 | | Up |
| 57 | Androstane-3,17-diol dipropionate | | 1.1288 | 3.506 | 3.77E-23 | | Up |
| 58 | Polyoxyethylene 40 monostearate | | 1.1138 | 3.7944 | 1.61E-31 | | Up |
| 59 | 1-(4-Amino-2-methylpyrimid-5-ylmethyl)-3-(beta-hydroxyethyl)-2-methylpyridinium | | 1.1032 | 11.101 | 3.04E-17 | | Up |
| 60 | N-stearoyl tyrosine | | 1.1004 | 9.3277 | 3.13E-20 | | Up |
| 61 | Lys Lys Arg Val | | 1.0986 | 12.378 | 2.50E-33 | | Up |
| 62 | Elaidic carnitine | | 1.0937 | 4.3693 | 2.30E-22 | | Up |
| 63 | Hydrolysis product of bussein | | 1.086 | 5.1681 | 1.08E-13 | | Up |
| 64 | Phosphodimethylethanolamine | | 1.0849 | 2.878 | 1.41E-18 | | Up |
| 65 | Ile Val Glu Lys Tyr | | 1.0692 | 14.895 | 5.71E-19 | | Up |
| 66 | Persicachrome | | 1.0306 | 2.9593 | 1.75E-19 | | Up |
| 67 | 5,7-Diethyl-9-methyl-3E,5E,7E,9E-tridecatetraene | | 1.0275 | 4.4662 | 3.18E-15 | | Up |
| 68 | PC(6:2(3E,5E)/14:2(11E,13E)) | | 1.0229 | 11.105 | 5.78E-29 | | Up |
| 69 | (4'R,6R,6'S,7S,8R)-6'-(2-Hydroxypentan-2-yl)-4',8-dimethyldecahydro-5H-spiro[indolizine-6,2'-pyran]-7,8-diol | | 1.0138 | 3.9758 | 1.88E-10 | | Up |
| 70 | Saccharopine | | 1.0123 | 3.7299 | 1.45E-15 | | Up |
| 71 | Glu Lys Asn Ile | | 1.0119 | 5.4455 | 7.29E-26 | | Up |
| 72 | Santene | | 1.0024 | 4.8229 | 4.69E-09 | | Up |
| 73 | DL-2-Aminooctanoic acid | | 1.0009 | 2.88 | 1.10E-16 | | Up |
| 74 | Allyl cinnamate | | 1.0007 | 2.9815 | 5.33E-09 | | Up |

**Table S7 Differentially expressed metabolites between BA and CS detected by negative ion mode acquisition**

| **NO.** | | **Metabolites** | **VIP**  **Value** | **Fold**  **Change** | ***P* Value** | **Regulation** |
| --- | --- | --- | --- | --- | --- | --- |
| 1 | Thr Cys Glu Pro Ile | | 2.8432 | 228.68 | 2.21E-28 | Up |
| 2 | Leu Asp Glu Cys | | 2.5876 | 390.61 | 2.18E-62 | Up |
| 3 | Sodium glycocholate | | 2.583 | 5.5029 | 3.63E-32 | Up |
| 4 | Isopropyl apiosylglucoside | | 1.9971 | 0.0061 | 2.49E-19 | Down |
| 5 | 3b,16a-Dihydroxyandrostenone sulfate | | 1.9923 | 8.3249 | 7.55E-22 | Up |
| 6 | Arg Cys Glu Arg | | 1.9199 | 5.4015 | 8.03E-25 | Up |
| 7 | 6, 8-Octadecadienoic acid | | 1.9091 | 3.0323 | 5.18E-23 | Up |
| 8 | Tyr Trp Trp Met | | 1.8686 | 0.0036 | 1.63E-27 | Down |
| 9 | Scillipheosidin 3-[glucosyl-(1-2)-rhamnoside] | | 1.834 | 1855.9 | 2.50E-11 | Up |
| 10 | Taurodeoxycholic acid | | 1.7475 | 3.775 | 1.33E-15 | Up |
| 11 | Lys Thr Thr Tyr | | 1.7408 | 5.9768 | 1.39E-18 | Up |
| 12 | (Z)-5-Hexadecenoic acid | | 1.6443 | 4.2576 | 3.18E-20 | Up |
| 13 | 2-C-Methyl-D-erythritol 4-phosphate | | 1.5686 | 3.1627 | 3.62E-18 | Up |
| 14 | Asp His Lys | | 1.4984 | 0.04357 | 3.44E-17 | Down |
| 15 | Raphanusamide | | 1.4244 | 2.5921 | 1.73E-11 | Up |
| 16 | 3-Hydroxycapric acid | | 1.3404 | 4.1486 | 1.07E-11 | Up |
| 17 | Isopalmitic acid | | 1.307 | 2.494 | 4.19E-19 | Up |
| 18 | Glu Glu Pro Trp | | 1.2783 | 44.879 | 4.07E-12 | Up |
| 19 | Dopamine 3-O-sulfate | | 1.2779 | 2.5068 | 2.72E-08 | Up |
| 20 | His Gly His Trp | | 1.2583 | 17.184 | 2.56E-18 | Up |
| 21 | Imazamethabenz | | 1.2097 | 15.919 | 5.14E-14 | Up |
| 22 | 3,9-Dimethyluric acid | | 1.1896 | 2.3225 | 1.76E-08 | Up |
| 23 | Metominostrobin | | 1.0782 | 13.227 | 1.11E-14 | Up |
| 24 | Desmethylmianserin glucuronide | | 1.0628 | 0.14907 | 7.88E-13 | Down |

**Table S8 The metabolites encompassed by the enriched pathways**

| **NO.** | | **Enrichment pathways** | **Metabolites** | **Group** |
| --- | --- | --- | --- | --- |
| 1 | Glycosylphosphatidylinositol-anchor biosynthesis | | 1-Phosphatidyl-D-myo-inositol | BA vs. NC |
| 2 | Citrate cycle (TCA cycle) | | Oxalosuccinic acid | BA vs. NC |
| 3 | Inositol phosphate metabolism | | 1-Phosphatidyl-D-myo-inositol | BA vs. NC |
| 4 | Sphingolipid metabolism | | Phytosphingosine | BA vs. NC |
| 5 | Glycerophospholipid metabolism | | 1-Phosphatidyl-D-myo-inositol | BA vs. NC |
| 6 | Phenylacetate Metabolism | | Alpha-N-Phenylacetyl-L-glutamine | CS vs. NC |
| 7 | Caffeine Metabolism | | 5-Acetylamino-6-formylamino  -3-methyluracil | CS vs. NC |
| 8 | Lysine Degradation | | Saccharopine | CS vs. NC |
| 9 | Lysine Degradation | | Saccharopine | BA vs. CS |
| 10 | Bile Acid Biosynthesis | | Taurodeoxycholic acid | BA vs. CS |


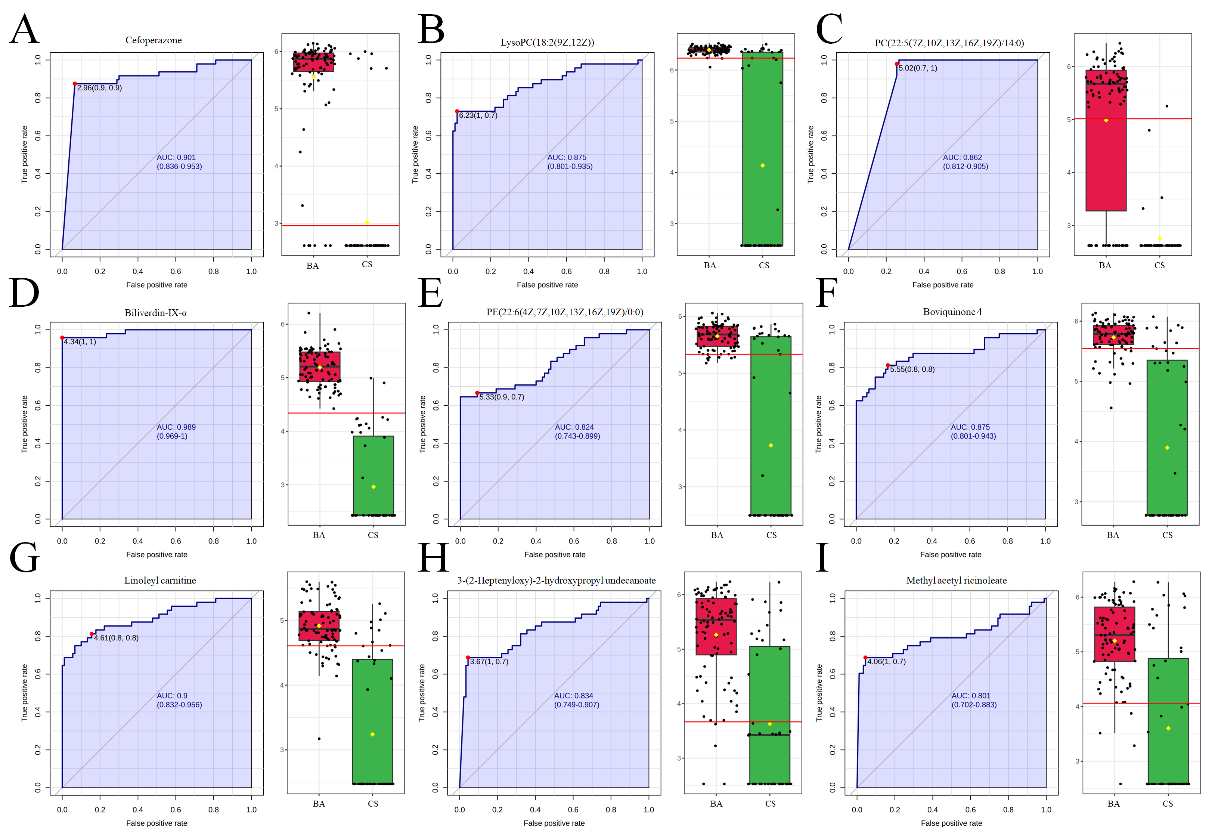


**Figure S4** The ROC curves and boxplots of 9 biomarkers in metabolomics in positive mode. The representative metabolic biomarker ROC curve and boxplot of (A) Cefoperazone, (B) LysoPC(18:2(9Z,12Z)), (C) PC(22:5(7Z,10Z,13Z,16Z,19Z)/14:0), (D) Biliverdin-IX-α, (E) PE(22:6(4Z,7Z,10Z,13Z,16Z,19Z)/0:0), (F) Boviquinone 4, (G) Linoleyl carnitine, (H) 3-(2-Heptenyloxy)-2-hydroxypropyl undecanoate, and (I) Methyl acetyl ricinoleate.

.


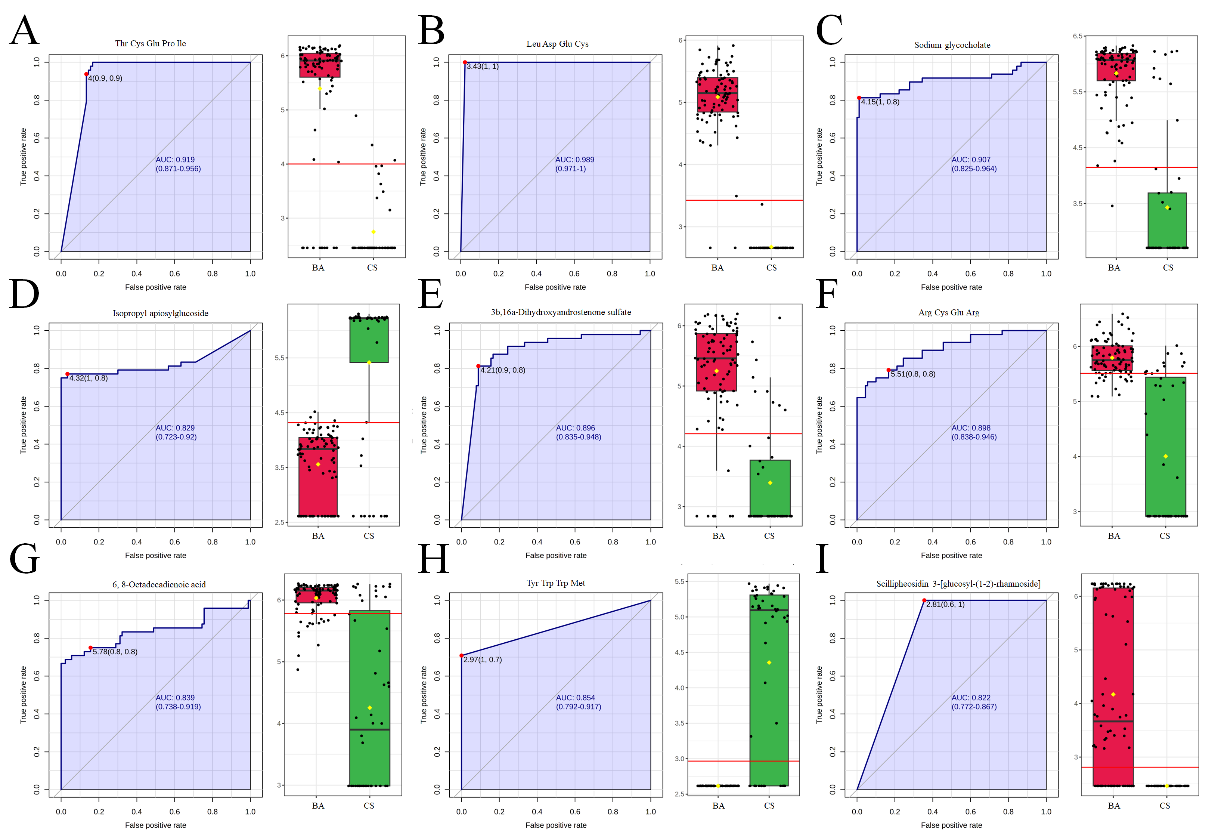


**Figure S5** The ROC curves and boxplots of 9 biomarkers in metabolomics in negative mode. The representative metabolic biomarker ROC curve and boxplot of (A) Thr Cys Glu Pro Ile, (B) Leu Asp Glu Cys, (C) Sodium glycocholate, (D) Isopropyl apiosylglucoside, (E) 3b,16a-Dihydroxyandrostenone sulfate, (F) Arg Cys Glu Arg, (G) 6, 8-Octadecadienoic acid, (H) Tyr Trp Trp Met, and (I) Scillipheosidin 3-[glucosyl-(1-2)-rhamnoside].
